# Pair-based likelihood approximations for stochastic epidemic models

**DOI:** 10.1093/biostatistics/kxz053

**Published:** 2019-12-06

**Authors:** Jessica E Stockdale, Theodore Kypraios, Philip D O’Neill

**Affiliations:** 1 Department of Mathematics, Simon Fraser University, 8888 University Drive, Burnaby, British Columbia V5A 1S6, Canada; 2 School of Mathematical Sciences, University of Nottingham, University Park, Nottingham NG7 2RD, UK

**Keywords:** Epidemic models, Likelihood approximation, Markov chain Monte Carlo methods, Stochastic epidemic models

## Abstract

Fitting stochastic epidemic models to data is a non-standard problem because data on the infection processes defined in such models are rarely observed directly. This in turn means that the likelihood of the observed data is intractable in the sense that it is very computationally expensive to obtain. Although data-augmented Markov chain Monte Carlo (MCMC) methods provide a solution to this problem, employing a tractable augmented likelihood, such methods typically deteriorate in large populations due to poor mixing and increased computation time. Here, we describe a new approach that seeks to approximate the likelihood by exploiting the underlying structure of the epidemic model. Simulation study results show that this approach can be a serious competitor to data-augmented MCMC methods. Our approach can be applied to a wide variety of disease transmission models, and we provide examples with applications to the common cold, Ebola, and foot-and-mouth disease.

## 1. Introduction

Mathematical models of infectious disease transmission are now routinely used as tools to assist with the analysis, prediction, and control of real-life epidemics. Such models may be deterministic or stochastic, are usually mechanistic in the sense that they seek to describe the process of disease spread between individuals, and invariably contain parameters such as infection rates that must be assigned values. The natural statistical problem that arises is to estimate the parameters of an epidemic model given observed data on one or more outbreaks of disease.

In this article, we focus exclusively on stochastic epidemic models. Fitting such a model to data in a frequentist or Bayesian framework requires evaluation of the likelihood of the observed data. In many situations, this is a non-standard problem because the infection process defined in the model is not observed in reality. Consequently, evaluation of the likelihood involves integrating over the set of all possible infection events that are compatible with the observed data, which is often a highly non-trivial exercise. Within the Bayesian framework, one solution is to use data augmentation, specifically including the unknown infection events as additional model parameters, which in turn leads to a computationally tractable likelihood. The posterior distribution of the model parameters given the data can then be explored via Markov chain Monte Carlo (MCMC) methods (Gibson and Renshaw, 1998; O’Neill and Roberts, 1999). However, such methods can struggle for large-scale problems, partly because the computational cost of evaluating the augmented-data likelihood increases, and partly because the missing data are strongly correlated to the model parameters which in turn creates mixing problems for the Markov chain. Although reparameterization techniques such as non-centering can help (Kypraios, 2007), it is still desirable to find alternative approaches.

In this article, we describe a method for approximating the likelihood of a partially observed stochastic epidemic model without the need for data augmentation. The key idea is to approximate the likelihood by a product of tractable terms that relate to either single individuals or pairs of individuals. Our approach is somewhat inspired by, but distinct from, a likelihood expression derived in Eichner and Dietz (2003) for a stochastic smallpox transmission model. As explained later, this expression is actually not the true likelihood for the model, but as shown in Stockdale *and others* (2017) it yields very similar parameter estimates to those obtained from a full data-augmented MCMC approach using the correct likelihood. Note also that our methods are unrelated to pair-approximation techniques used in the analysis of deterministic epidemic models (Keeling *and others*, 1997).

Our main aim is to explore the potential utility of using approximate likelihood methods in fitting stochastic epidemic models to data. The work presented here is in some sense preliminary, since there are many possible future directions that could be taken, but also contains many promising results. The article is structured as follows. Section 2 defines the epidemic model of interest and associated notation. The likelihood approximations are developed in Section 3 and illustrated via three applications in Section 4. Brief conclusions are given in Section 5. Details of proofs and results from an extensive simulation study can be found in the Supplementary material available at *Biostatistics* online.

## 2. Preliminary material

For ease of exposition, we shall describe likelihood approximations for a specific stochastic epidemic model defined below. However, similar approximations can be derived for more complex models, as illustrated in Section 4.

### 2.1. Stochastic epidemic model

The following epidemic model generalizes the well-known general stochastic epidemic (see e.g. Bailey, 1975; Andersson and Britton, 2000) so that the infection rate between a pair of individuals is allowed to depend on who the individuals are, and infectious period distributions can vary between individuals.

Consider a population consisting of $N$ individuals, labeled $1, \ldots, N$. At any time, each individual is either *susceptible*, meaning they are capable of contracting the disease in question, *infective*, meaning that they have the disease and can infect others, or *removed*, meaning that they are no longer able to infect others and cannot be re-infected. The precise interpretation of the removed state depends on the disease in question, examples including isolation, recovery, or death. Initially, the population is entirely susceptible apart from a few infective individuals. Each individual who becomes infective remains so for a period of time known as the infectious period. The infectious period of individual $j$ is distributed according to some pre-specified random variable $I_j$. At the end of its infectious period, an individual becomes removed. The infectious periods of different individuals are assumed to be independent.

During its infectious period, a given infective individual $j$ has contacts with susceptible individual $k$ at times given by the points of a Poisson process of rate $\beta_{jk}$. All such Poisson processes are mutually independent and independent of the infectious periods. Any contact that occurs results in the susceptible individual $k$ immediately becoming infective. We define the *infectious pressure* acting on susceptible $k$ at time $t$ as the hazard rate of infection at time $t$, in other words $\Sigma \beta_{jk}$ where the sum is over all individuals $j$ who are infective at time $t$.

The epidemic continues until there are no infectives remaining. Thus at the end of the epidemic, each initially susceptible individual is either still susceptible, or removed. Finally, the population is assumed to be closed in the sense that no individuals may enter or leave during the epidemic.

The model defined above is rather general and contains $N(N-1)$ infection rate parameters corresponding to all possible choices of the ordered pair $(j,k)$, $j \neq k$. For specific modeling situations we usually use a model with fewer parameters, which can be obtained by making suitable assignments for the $\beta_{jk}$ and the parameters governing the infectious period distributions. Examples include the general stochastic epidemic, i.e. the standard homogeneously mixing Susceptible-Infective-Removed (SIR) model, multi-type models, models with two or more levels of mixing, and spatial models.

### 2.2. Notation, data, and likelihood

In real-life epidemics, the actual transmission process of infection between individuals is rarely observed. We therefore suppose henceforth that the observed data consist of the times of all removal events, and re-label members of the population such that individuals $1, \ldots, n$ are those who are ultimately removed, and $n+1, \ldots, N$ are those (if any) who remain susceptible, where $n \leq N$. We are thus implicitly assuming that each removal event in the model corresponds to a real-life observable event such as the appearance of symptoms in an individual, and furthermore that the individual is then unable to infect others, perhaps due to isolation. We are also assuming that the epidemic has come to an end, so that there are no unobserved removals.

For $j = 1, \ldots, n$ let $r_j$ denote the time of removal of individual $j$, with the convention that $r_j = \infty$ if $j$ is never removed, i.e. if $j > n$. Similarly define $i_j$ as the time at which $j$ becomes infected, with $i_j = \infty$ if this never occurs. We assume that there is a single initial infective $\alpha$, so that $\alpha \in \left\{ 1, \ldots, n \right\}$, but we do not assume that $\alpha$ is known from the data. The assumption of a single initial infective is not necessary, but simplifies our exposition and is often realistic in practice. Let ${r} = (r_1, \ldots, r_n)$ and ${i} = (i_1, \ldots, i_{\alpha-1}, i_{\alpha+1}, \ldots, i_n)$, so that ${i}$ contains all infection times other than $i_\alpha$. Denote by ${\beta} = \left\{ \beta_{jk} : 1 \leq j,k \leq N, j \neq k \right\}$ the set of infection rate parameters in the model.

Let ${\theta}_j$ denote the parameter vector of the infectious period distribution for individual $j$, $j=1, \ldots, n$. Our main focus will be upon the cases where the infectious periods are either exponential or Erlang with known shape parameter, where in both cases ${\theta}_j$ is one-dimensional. Finally, let ${\theta} = \left\{ {\theta}_j : 1 \leq j \leq n \right\}$ denote the set of infectious period distribution parameters.

Our objective is to make inference for the parameters ${\beta}$ and ${\theta}$ given the data ${{r}}$, assuming the population size $N$ is known. Any likelihood-based approach therefore requires evaluation of the likelihood $\pi ( {r} \,{|}\, {\beta}, {\theta})$ but calculating this quantity is highly computationally expensive. The reason for this is that any such calculation implicitly or explicitly involves integrating over all possible values of the unknown infection times, the set of which is non-trivial due to the constraints that (i) $i_j < r_j$ for $j = 1, \ldots, n$ and (ii) at any time during the epidemic, there cannot be more removals than infections (see e.g. Clancy and O’Neill, 2008).

One solution to this problem is to instead work with the tractable augmented likelihood $\pi( {i}, {r} \,{|}\, {\beta}, {\theta}, \alpha, i_\alpha)$, given explicitly below. For instance, in a Bayesian framework the unknown infection times can be incorporated as extra parameters and an MCMC algorithm can be used to sample from the resulting posterior distribution, as described in O’Neill and Roberts (1999). Our objective here, however, is to find a way of approximating the likelihood $\pi ( {r} \,{|}\, {\beta}, {\theta})$ that avoids any data augmentation.

## 3. Pair-based likelihood approximation

### 3.1. Notation and augmented likelihood

Consider an individual $j$ who becomes infected at time $i_j$ and removed at time $r_j$. Define

$$\begin{eqnarray*}
\psi_j & = & P(\mbox{$j$ avoids infection until time $i_j$}),\\
\chi_j & = & \mbox{infectious pressure acting on $j$ as they become infected},\\
\mbox{and} \; \; \phi_j & = & P(\mbox{$j$ fails to infect any of the $N-n$ never-infected individuals}).
\end{eqnarray*}$$

It follows from the definition of the epidemic model that

(1) \begin{eqnarray*} \psi_j & = & \exp \left( - \sum_{ \substack{k=1\\k \neq j}}^n \beta_{kj} \tau_{kj} \right)\!, \label{eq:psi}\\ \end{eqnarray*}

(2) \begin{eqnarray*} \chi_j & = & \sum_{ \substack{k=1\\k \neq j}}^n \beta_{kj} 1_{ \left\{ i_k < i_j < r_k \right\} }, \label{eq:chi}\\ \end{eqnarray*}

(3) \begin{eqnarray*} \mbox{and} \; \; \phi_j & = & \exp \left( - \sum_{k=n+1}^N \beta_{jk} (r_j - i_j) \right) = \exp \left( - (r_j - i_j) B_j \right)\!, \label{eq:phi} \end{eqnarray*}

say, where, with $\wedge$ denoting minimum,

(4) \begin{equation*} \label{eq:taukj} \tau_{kj} = r_k \wedge i_j - i_k \wedge i_j \end{equation*}

is the length of time during which $k$ is infective and $j$ susceptible, and $1_A$ denotes the indicator function of the event $A$. Note that the summation terms in (1) and (2) could both be written without excluding $k \neq j$, since both $\tau_{jj}$ and $1_{ \left\{ i_j < i_j < r_j \right\} }$ are zero. However, when translating formulae into computer code it is helpful to know what can be excluded from sums or products, so our derivations will make this explicit.

For ease of exposition, we assume that the infectious period distributions are continuous and let $f_j$ denote the probability density function of $I_j$; the arguments below also hold without this assumption. The augmented likelihood of all infection and removal events may be written as

(5) \begin{equation*} \pi( {i}, {r} \,{|}\, {\beta}, {\theta}, \alpha, i_\alpha) = \left\{ \prod_{\substack{j=1\\j \neq \alpha}}^n \chi_j \psi_j \phi_j f_j(r_j - i_j \,{|}\, {\theta}_j) \right\} \phi_\alpha f_\alpha(r_\alpha - i_\alpha \,{|}\, {\theta}_\alpha). \label{eq:auglike} \end{equation*}

We now briefly explain (5); a more detailed explanation for the special case of the general stochastic epidemic can be found in Andersson and Britton (2000). The product term accounts for each ever-infected individual $j$ (other than $\alpha$) avoiding infection until time $i_j$, becoming infected at time $i_j$, remaining infective until time $r_j$, and whilst infective failing to infect the $N-n$ individuals who avoid infection entirely. Note that the probability of $j$ failing to infect another individual $k$, prior to $k$’s own infection at time $i_k$, will be accounted for in $\psi_k$. Finally, the corresponding likelihood contribution for $\alpha$ is similar to that of $j$, but does not account for how $\alpha$ became infected since the epidemic model only describes events after the initial infection.

Equation (5) is not the only way to write the likelihood: for example, all of the $\psi_j$ and $\phi_j$ terms could be combined together to give the total probability of individuals avoiding infection during the epidemic. However, (5) is in a form suitable for our purposes. Note also that the $\psi_j$ and $\chi_j$ terms appearing in the product do not only depend on individual $j$, but also on the infection and removal times of other individuals.

### 3.2. Derivation of the pair-based approximation

We now derive an approximation to the desired likelihood $\pi ( {r} \,{|}\, {\beta}, {\theta})$. First note that

$$
\pi ( {r} \,{|}\, {\beta}, {\theta}) = \int \pi( {i}, {r} \,{|}\, {\beta}, {\theta}, \alpha, i_\alpha) \pi(i_\alpha,\alpha) {\rm d}{i} \; {\rm d}i_\alpha \; {\rm d}\alpha,
$$

where the integral represents integration over ${i}$, $i_\alpha,$ and summation over $\alpha$, and where $(i_\alpha,\alpha)$ is assumed to be independent of $({\beta}, {\theta})$*a priori*. Thus,

(6) \begin{eqnarray*} \pi ( {r} \,{|}\, {\beta}, {\theta}) & = & \sum_{\alpha=1}^n \pi(\alpha) \int \pi( {i}, {r} \,{|}\, {\beta}, {\theta}, \alpha, i_\alpha) \pi(i_\alpha \,{|}\, \alpha) {\rm d}{i} \; {\rm d}i_\alpha \nonumber \\ & = & \sum_{\alpha=1}^n \pi(\alpha) \int \left\{ \prod_{\substack{j=1\\j \neq \alpha}}^n \chi_j \psi_j \phi_j \right\} \phi_\alpha \pi(i_\alpha \,{|}\, \alpha) \left\{ \prod_{j =1}^n f_j(r_j - i_j \,{|}\, {\theta}_j) \right\} {\rm d}{i} \; {\rm d}i_\alpha \nonumber \\ & = & \sum_{\alpha=1}^n \pi(\alpha) \int \left\{ \prod_{\substack{j=1\\j \neq \alpha}}^n \chi_j \psi_j \right\} \pi(i_\alpha \,{|}\, \alpha) \left\{ \prod_{j =1}^n \phi_j f_j(r_j - i_j \,{|}\, {\theta}_j) \right\} {\rm d}{i} \; {\rm d}i_\alpha, \label{eq:PBLA1} \end{eqnarray*}

where $\pi(\alpha)$ denotes the prior probability that $\alpha$ is the initial infective. For $j = 1, \ldots, n$,

$$\begin{eqnarray*}
\phi_j f_j(r_j - i_j \,{|}\, {\theta}_j) & = & \exp \left( - (r_j - i_j) B_j \right) f_j(r_j - i_j \,{|}\, {\theta}_j) \\
& = & a({\theta}, B_j) g_j(r_j - i_j \,{|}\, {\theta}_j),
\end{eqnarray*}$$

say, where $g_j$ is the probability density function defined for $x > 0$ by

(7) \begin{equation*} g_j(x \,{|}\, {\theta}_j) = \frac{\exp(-xB_j) f_j(x \,{|}\, {\theta}_j)}{\int \exp(-xB_j) f_j(x \,{|}\, {\theta}_j) \; {\rm d}x } = \frac{\exp(-xB_j) f_j(x \,{|}\, {\theta}_j)}{a({\theta}_j, B_j)}, \label{eq:gj} \end{equation*}

and $g_j(x \,{|}\, {\theta}_j) = 0$ for $x \leq 0$. Thus, $a({\theta}_j, B_j)$ is the moment generating function of the infectious period $I_j$ evaluated at $B_j$. Substituting into (6) yields

(8) \begin{eqnarray*} \pi ( {r} \,{|}\, {\beta}, {\theta}) & = & \left\{ \prod_{j=1}^n a({\theta}_j, B_j) \right\} \sum_{\alpha=1}^n \pi(\alpha) \int \left\{ \prod_{\substack{j=1\\j \neq \alpha}}^n \chi_j \psi_j \right\} \pi(i_\alpha \,{|}\, \alpha) \left\{ \prod_{j =1}^n g_j(r_j - i_j \,{|}\, {\theta}_j) \right\} {\rm d}{i} \; {\rm d}i_\alpha \nonumber \\ & = & \left\{ \prod_{j=1}^n a({\theta}_j, B_j) \right\} \sum_{\alpha=1}^n \pi(\alpha) \mathbb{E}_{{g}} \left[\pi(i_\alpha \,{|}\, \alpha) \prod_{\substack{j=1\\j \neq \alpha}}^n \chi_j \psi_j \right], \label{eq:PBLA2} \end{eqnarray*}

where $\mathbb{E}_{{g}}$ denotes expectation of $(r_1-i_1, \ldots, r_n-i_n)$ with respect to the product density

(9) \begin{equation*} {g}(x_1, \ldots, x_n \,{|}\, {\theta}) = \prod_{j=1}^n g_j(x_j\,{|}\, {\theta}_j). \label{eq:gproduct} \end{equation*}

Note that here we regard ${r}$ as fixed and the infection times as random variables, and thus sampling from ${g}$ essentially generates a sample from $({i}, i_\alpha)$. Evaluating the required likelihood thus requires evaluation of the expectation term in (8).

Before turning to approximations we make some remarks about the exact equation (8). First, (6) can clearly be written as an expectation with respect to the product density obtained by multiplying the $f_j$ terms together. The advantage of the approach leading to (8) is that the $\phi_j$ terms are absorbed into the expectation, and thus we do not need to evaluate or approximate them. Second, let $\mathcal{I}$ denote the set of values of $({i}, i_\alpha)$ such that the term inside the expectation in (8) is non-zero. Although the expectation is taken with respect to independent random variables, $\mathcal{I}$ is complicated in structure, which makes analytical progress difficult. Finally, a random sample from ${g}$ is not guaranteed to lie inside $\mathcal{I}$, which makes standard Monte Carlo estimation inefficient. An importance sampling estimator could be constructed, although it is not obvious how to construct an efficient proposal distribution for the infection times. We therefore proceed via an approximation in which we assume independence of the product terms in the expectation term in (8), as follows.

First, we assume that

(10) \begin{equation*}\label{eq:PBLA3} \mathbb{E}_{{g}} \left[\pi(i_\alpha \,{|}\, \alpha) \prod_{\substack{j=1\\j \neq \alpha}}^n \chi_j \psi_j \right] \approx \mathbb{E}_{{g}} \left[ \pi(i_\alpha \,{|}\, \alpha) \right] \prod_{\substack{j=1\\j \neq \alpha}}^n \mathbb{E}_{{g}} \left[ \chi_j \psi_j \right]. \end{equation*}

Evaluation of the first expectation in (10) depends on the choice of prior density $\pi(i_\alpha \,{|}\, \alpha)$, but is often straightforward in practice. For the second expectation, first note that

$$
\psi_j = \exp \left( - \sum_{\substack{l=1\\l \neq j}}^n \beta_{lj} \tau_{lj} \right) = \prod_{\substack{l=1\\l \neq j}}^n \exp ( - \beta_{lj} \tau_{lj} )
= \prod_{\substack{l=1\\l \neq j}}^n \psi_{jl},
$$

say. Then,

(11) \begin{eqnarray*} \mathbb{E}_{g} \left[ \chi_j \psi_j \right] & = & \sum_{\substack{k=1\\k \neq j}}^n \beta_{kj} \mathbb{E}_{{g}} \left[ 1_{ \left\{ i_k < i_j < r_k \right\} } \psi_j \right] \nonumber \\ & = & \sum_{\substack{k=1\\k \neq j}}^n \beta_{kj} \mathbb{E}_{{g}} \left[ 1_{ \left\{ i_k < i_j < r_k \right\} } \prod_{\substack{l=1\\l \neq j}}^n \psi_{jl} \right] \nonumber \\ & = & \sum_{\substack{k=1\\k \neq j}}^n \beta_{kj} \mathbb{E}_{{g}} \left[ 1_{ \left\{ i_k < i_j < r_k \right\} } \psi_{jk} \prod_{\substack{l=1\\l \neq j,k}}^n \psi_{jl} \right] \nonumber \\ & \approx & \sum_{\substack{k=1\\k \neq j}}^n \beta_{kj} \mathbb{E}_{{g}} \left[ 1_{ \left\{ i_k < i_j < r_k \right\} } \psi_{jk} \right] \prod_{\substack{l=1\\l \neq j,k}}^n \mathbb{E}_{{g}} \left[ \psi_{jl} \right], \label{eq:PBLA4} \end{eqnarray*}

where (11) only contains terms that concern pairs of individuals. For computational purposes, it is useful to re-write (11) as

(12) \begin{equation*} \mathbb{E}_{{g}} \left[ \chi_j \psi_j \right] \approx \left\{ \prod_{\substack{l=1\\l \neq j}}^n \mathbb{E}_{{g}} \left[ \psi_{jl} \right] \right\} \sum_{\substack{k=1\\k \neq j}}^n \beta_{kj} \mathbb{E}_{{g}} \left[ 1_{ \left\{ i_k < i_j < r_k \right\} } \psi_{jk} \right] \left( \mathbb{E}_{{g}} \left[ \psi_{jk} \right] \right)^{-1}, \label{eq:PBLA5} \end{equation*}

to avoid computing the product terms in (11) separately for each term in the sum.

Definition 1 We refer to the approximation arising from equations (8), (10), and (12) as the *standard* pair-based likelihood approximation (PBLA).

To evaluate (12), we need to compute, for $j \neq k$,

(13) \begin{equation*} \mathbb{E}_{{g}} \left[ \psi_{jk} \right] = \mathbb{E}_{g_j,g_k} \left[ \exp ( - \beta_{kj} \tau_{kj} ) \right]\!,\label{eq:psi_jk} \end{equation*}

which is the probability that $j$ avoids infection from $k$ while $k$ is infective and $j$ susceptible, and the related quantity

(14) \begin{equation*} \mathbb{E}_{{g}} \left[ 1_{ \left\{ i_k < i_j < r_k \right\} } \psi_{jk} \right] = \mathbb{E}_{g_j,g_k} \left[ 1_{ \left\{ i_k < i_j < r_k \right\} } \exp ( - \beta_{kj} \tau_{kj} ) \right]\!.\label{eq:1psi_jk} \end{equation*}

Explicit expressions for (13) and (14) for the case of exponential or Erlang infectious period distributions are given below in Sections 3.4 and 3.5, respectively.

Finally, it is important to note that the standard approximation, and those described below, become exact in the special case where infectious periods are non-random, since the expectations are redundant. This in turn suggests that the less variability an infectious period distribution has, the more accurate the approximation will be.

### 3.3. Alternative approximations

The derivation of (12) is clearly not the only way to derive an approximate likelihood, and here we mention some alternatives.

#### 3.3.1. Use $f_j$ for expectations

As mentioned previously one could proceed without introducing the change of density from $f_j$ to $g_j$, so that (8) becomes

(15) \begin{equation*} \label{eq:fapprox} \pi ( {r} \,{|}\, {\beta}, {\theta}) = \sum_{\alpha=1}^n \pi(\alpha) \mathbb{E}_{{f}} \left[\phi_{\alpha} \pi(i_\alpha \,{|}\, \alpha) \prod_{\substack{j=1\\j \neq \alpha}}^n \chi_j \psi_j \phi_j \right]\!, \end{equation*}

where the expectation is with respect to the product density $\prod_{j=1}^n f_j(x_j \,{|}\, {\theta}_j)$ (cf. (9)). Following the arguments above leads naturally to terms of the form

$$\begin{eqnarray*}
\mathbb{E}_{{f}} [ \chi_j \psi_j \phi_j ] & \approx & \mathbb{E}_{{f}} [ \chi_j \phi_j ] \mathbb{E}_{{f}} [ \psi_j ] \\
& \approx & \mathbb{E}_{{f}} [ \chi_j \phi_j ] \prod_{\substack{k=1\\k \neq j}}^n \mathbb{E}_{{f}} [ \psi_{jk} ],
\end{eqnarray*}$$

evaluation of which requires the equivalent of (13) with ${f}$ replacing ${g}$, and

$$
\mathbb{E}_{{f}} [ \chi_j \phi_j ] = \mathbb{E}_{{f}} \left[ 1_{ \left\{ i_k < i_j < r_k \right\} } \exp \left( - (r_j - i_j) B_j \right) \right].
$$

#### 3.3.2. Separate all $\chi$ and $\psi$ terms

We could write

$$\begin{eqnarray*}
\mathbb{E}_{{g}} \left[ \chi_j \psi_j \right] & \approx & \mathbb{E}_{{g}} \left[ \psi_j \right] \mathbb{E}_{{g}} \left[ \chi_j \right]\\
& \approx & \left\{ \prod_{\substack{l=1\\l \neq j}}^n
\mathbb{E}_{{g}} \left[ \psi_{jl} \right] \right\}
\sum_{\substack{k=1\\k \neq j}}^n \beta_{kj} \mathbb{E}_{{g}} \left[
1_{ \left\{ i_k < i_j < r_k \right\} } \right]\!,
\end{eqnarray*}$$

following the arguments leading to (12). We found that this formulation is numerically fairly similar to (12), although it involves marginally more approximation because the indicator function and $\psi_{jk}$ terms are separated.

#### 3.3.3. Approximate product of $\psi$ terms

We could attempt to approximate the entire product of $\psi_j$ terms. The expectation in (8) may be approximated by

(16) \begin{equation*} \label{eq:psi_product} \mathbb{E}_{{g}} \left[\pi(i_\alpha \,{|}\, \alpha) \prod_{\substack{j=1\\j \neq \alpha}}^n \chi_j \psi_j \right] \approx \mathbb{E}_{{g}} \left[ \pi(i_\alpha \,{|}\, \alpha) \right] \left\{ \prod_{\substack{j=1\\j \neq \alpha}}^n \mathbb{E}_{{g}} \left[ \chi_j \right] \right\} \left\{ \mathbb{E}_{{g}} \left[ \prod_{\substack{j=1\\j \neq \alpha}}^n \psi_j \right] \right\}, \end{equation*}

where as above we have

(17) \begin{equation*} \label{eq:chiapprox} \mathbb{E}_{{g}} \left[ \chi_j \right] = \sum_{\substack{k=1\\k \neq j}}^n \beta_{kj} \mathbb{E}_{{g}} \left[ 1_{ \left\{ i_k < i_j < r_k \right\} } \right], \end{equation*}

while

(18) \begin{equation*} \label{eq:doublesum} \mathbb{E}_{{g}} \left[ \prod_{\substack{j=1\\j \neq \alpha}}^n \psi_j \right] = \mathbb{E}_{{g}} \left[ \exp \left( - \sum _{\substack{j=1\\j \neq \alpha}}^n \sum_{ \substack{k=1\\k \neq j}}^n \beta_{kj} \tau_{kj} \right) \right]. \end{equation*}

In Section 3.4.2 below, we describe methods that exploit (18) for the case where $\beta_{kj} = \beta/N$ and infectious periods are exponentially distributed.

#### 3.3.4. Eichner and Dietz approximation

Eichner and Dietz (2003) define a stochastic model for smallpox transmission and give a likelihood expression which is used for maximum likelihood estimation of the model parameters. Although not presented as such, their expression is actually an approximation to the true likelihood. For our model, their method is as follows. For $j=1, \ldots, n$ define

$$
\lambda_j(u) = \sum_{ \substack{k=1\\k \neq j}}^n \beta_{kj} 1_{ \left\{ i_k < u < r_k \right\} }
$$

as the infectious pressure acting on individual $j$ at time $u$. Note that $\chi_j = \lambda_j(i_j)$. The Eichner–Dietz (ED) likelihood approximation is

(19) \begin{eqnarray*} \pi( {r} \,{|}\, {\beta}, {\theta}) \approx \pi_{\rm ED}( {r} \,{|}\, {\beta}, {\theta}) & = & \left\{ \prod_{j=1}^n \left( \int_{- \infty}^{r_j} \mathbb{E}[\lambda_j(i_j)] \exp \left( - \int_{-\infty}^{i_j} \mathbb{E}[\lambda_j(s)] \; {\rm d}s \right) f_j(r_j - i_j\,{|}\,{\theta}_j) \; {\rm d}i_j \right) \right\} \nonumber\\ && \times \prod_{j=n+1}^N \exp \left( - \int_{-\infty}^{r_n} \mathbb{E}[\lambda_j(s)] \; {\rm d}s \right), \label{eq:EDapprox} \end{eqnarray*}

where

(20) \begin{equation*} \label{eq:EDapproxlambda} \mathbb{E}[\lambda_j(u)] = \sum_{ \substack{k=1\\k \neq j}}^n \beta_{kj} P( i_k < u < r_k ) = \sum_{ \substack{k=1\\k \neq j}}^n \beta_{kj} 1_{ \left\{ u < r_k \right\}} \int_{r_k-u}^{\infty} f_k(s\,{|}\, {\theta}_k) \; {\rm d}s, \end{equation*}

since $r_k - i_k$ has density $f_k$.

The ED approximation could be derived as follows. The starting point is to assume that the likelihood takes the form

(21) \begin{equation*} L_{\rm ED} = \mathbb{E}_{{f}} \left[ \prod_{j=1}^n \chi_j \psi_j \phi_j \right], \label{eq:ED1} \end{equation*}

which bears some resemblance to the exact expression at (15) (e.g. by setting $\pi(\alpha) = 1_{ \left\{ \alpha = 1 \right\} }$), but differs because the initial infective is not explicitly included. Now,

$$
\prod_{j=1}^n \phi_j = \exp \left( - \sum_{j=1}^n \sum_{k=n+1}^N \beta_{jk} (r_j - i_j) \right) =
\prod_{k=n+1}^N \exp \left( - \sum_{j=1}^n \beta_{jk} \tau_{jk} \right)
= \prod_{k=n+1}^N \tilde{\phi}_k,
$$

say, since $\tau_{jk} = (r_j - i_j)$ for $k > n$. Note that $\tilde{\phi}_k$ is the probability that individual $k$ avoids infection throughout the epidemic. Thus (21) may be written as

(22) \begin{eqnarray*} L_{ED} & = & \mathbb{E}_{{f}} \left[ \left\{ \prod_{j=1}^n \chi_j \psi_j \right\} \left\{ \prod_{k=n+1}^N \tilde{\phi}_k \right\} \right] \nonumber \\ & \approx & \left\{ \prod_{j=1}^n \mathbb{E}_{{f}} [\chi_j \psi_j ]\right\} \left\{ \prod_{k=n+1}^N \mathbb{E}_{{f}} [\tilde{\phi}_k ]\right\} \nonumber \\ & \approx & \left\{ \prod_{j=1}^n \mathbb{E}_{f_j} \left[ \mathbb{E}_{{f}} [\chi_j \,{|}\, i_j ] \mathbb{E}_{{f}} [\psi_j \,{|}\, i_j ] \right] \right\} \left\{ \prod_{k=n+1}^N \mathbb{E}_{{f}} [\tilde{\phi}_k ]\right\}, \label{eq:ED2} \end{eqnarray*}

where $\mathbb{E}_{f_j}$ denotes expectation of $i_j$ with respect to $f_j(r_j - i_j \,{|}\, {\theta}_j)$. The expectations in (22) are then evaluated using further approximations as shown in (19).

The main difficulty with (19) is that, in practice, it involves numerical integration. As shown below, even for the general stochastic epidemic the integral appears to be analytically intractable. However, conditioning on $i_j$ is an attractive feature of the ED approximation, since it removes one of the sources of approximation in the pair-based methods.

### 3.4. Exponential infectious periods

In this section, we assume that for $j = 1, \ldots, n$ the infectious period random variable $I_j$ is exponential with rate parameter ${\theta}_j = \gamma_j$, denoted by $I_j \sim Exp(\gamma_j)$. Such models, particularly the case where $\gamma_j = \gamma$ for all $j$, appear frequently in the epidemic modeling literature, largely because of their relative mathematical tractability.

Let $x>0$. Since $f_j(x\,{|}\,{\theta}_j) = \gamma_j \exp(- \gamma_j x)$ then for $j = 1, \ldots, n$, (7) gives

$$
g_j(x \,{|}\, {\theta}_j) = \frac{\gamma_j \exp( - (\gamma_j + B_j)x)}{\gamma_j/(\gamma_j+B_j)} = \delta_j \exp( - \delta_j x),
$$

say, so that $g_j$ is the probability density function of an exponential random variable with rate $\delta_j = \gamma_j + B_j$, and

$$
a({\theta}_j, B_j) = a(\gamma_j, B_j) = \gamma_j/(\gamma_j+B_j) = \gamma_j/\delta_j.
$$

#### 3.4.1. The standard pair-based approximation with exponential infectious periods

We require expressions for (13) and (14). Recall from (4) that $\tau_{kj} = r_k \wedge i_j - i_k \wedge i_j$.

Lemma 1 Let $1 \leq j,k \leq n$ with $j \neq k$, and $\beta > 0$. Then

$$\begin{eqnarray*}
&&{
\mathbb{E}_{g_j,g_k} \left[ \exp ( - \beta \tau_{kj} ) \right] }\\
&& = \left\{
\begin{array}{ll}
1 - \beta \delta_j \left\{ (\delta_j+\delta_k)(\beta+\delta_k) \right\}^{-1} \exp (- \delta_k (r_k - r_j)) & \mbox{if $r_j < r_k$},\\
\delta_k(\beta+ \delta_k)^{-1} + \beta \delta_k \left\{ (\delta_j+\delta_k)(\beta+\delta_k) \right\}^{-1} \exp (- \delta_j (r_j - r_k)) & \mbox{if $r_j > r_k$},
\end{array}\right.\\
&&{
\mathbb{E}_{g_j,g_k} \left[ 1_{ \left\{ i_k < i_j < r_k \right\} } \exp ( - \beta \tau_{kj} ) \right] }\\
&& = \left\{
\begin{array}{ll}
\delta_j \delta_k \left\{ (\delta_j+\delta_k)(\beta+\delta_k) \right\}^{-1} \exp (- \delta_k (r_k - r_j)) & \mbox{if $r_j < r_k$},\\
\delta_j \delta_k \left\{ (\delta_j+\delta_k)(\beta+\delta_k) \right\}^{-1} \exp (- \delta_j (r_j - r_k)) & \mbox{if $r_j > r_k$}.
\end{array}
\right.
\end{eqnarray*}$$

Lemma 1 can be proved either by direct calculation or by probability arguments; see the Supplementary material available at *Biostatistics* online for details.

#### 3.4.2. Approximations for the general stochastic epidemic

Suppose now that for $1 \leq j,k \leq N$, $\beta_{kj} = \beta/N$ and $\gamma_j =\gamma$, so that the epidemic model is the general stochastic epidemic. From (3) we have $B_j = (N-n)\beta/N$, and thus $\delta_j = \gamma + (N-n)\beta/N = \delta$, say. This in turn leads to some simplifications in the expressions in Lemma 1 for the standard approximation.

We now focus on approximations that involve the product of $\psi$ terms. Note that (18) becomes

(23) \begin{equation*} \label{eq:doublesum2} \mathbb{E}_{{g}} \left[ \prod_{\substack{j=1\\j \neq \alpha}}^n \psi_j \right] = \mathbb{E}_{{g}} \left[ \exp \left( - (\beta/N) \sum_{\substack{j=1\\j \neq \alpha}}^n \sum_{ \substack{k=1\\k \neq j}}^n \tau_{kj} \right) \right]. \end{equation*}

Recall from (4) that $\tau_{kj}$ is the length of time during which $k$ is able to infect $j$. Thus for a given set of infection times, one of $\tau_{kj}$ and $\tau_{jk}$ is zero. To exploit this dependency, we rewrite the double sum in (23), also using the facts that $\tau_{k \alpha} = \tau_{jj} = 0$, to give

(24) \begin{equation*} \label{eq:doublesumomega} \sum_{\substack{j=1\\j \neq \alpha}}^n \sum_{ \substack{k=1\\k \neq j}}^n \tau_{kj} = \sum_{j=1}^{n-1} \sum_{k = j+1}^n (\tau_{kj} + \tau_{jk}) = \sum_{j=1}^{n-1} \sum_{k = j+1}^n \omega_{jk} = X, \end{equation*}

say, where, since $r_j < r_k$ for $j < k$, we have

(25) \begin{equation*} \label{eq:omega} \omega_{jk} = \left\{ \begin{array}{ll} i_j - i_k & \mbox{if $i_k < i_j$},\\ i_k - i_j & \mbox{if $i_j < i_k < r_j$},\\ r_j - i_j & \mbox{if $i_k > r_j$}. \end{array} \right. \end{equation*}

Note that $\omega_{jk}$ is the length of time that $j$ exerts infectious pressure on $k$, or vice versa. We are thus concerned with the behavior of $X$ given that $r_1 - i_1, \ldots, r_n - i_n$ are independent exponential distributions with parameter $\delta$. The following result, proved in the Supplementary material available at *Biostatistics* online, provides an explicit distribution for the total infectious pressure time among any subset of individuals in $\left\{ 1, \ldots, n \right\}$.

Lemma 2 Let $\mathcal{K}$ be any subset of $\left\{ 1, \ldots, n \right\}$ with $K \geq 2$ elements. If $\left\{ r_j - i_j : j \in \mathcal{K} \right\}$ is a set of independent $Exp(\delta)$ random variables, then

$$
\sum_{\substack{j, k \in \mathcal{K}\\ j < k}} \omega_{jk} \sim \sum_{j=1}^{K-1} Y_j,
$$

where $Y_j \sim Exp(\delta/j)$ and $Y_1, \ldots, Y_{K-1}$ are independent.

Setting $K=2$ in Lemma 2 yields that $\omega_{jk} \sim Exp(\delta)$ for any $j \neq k$, and setting $K=n$ yields an explicit distribution for $X$ in (24). Furthermore, (23) and Lemma 2 yield the result

$$
\mathbb{E}_{{g}} \left[ \prod_{\substack{j=1\\j \neq \alpha}}^n
\psi_j \right] = \prod_{j=1}^{n-1} \left( \frac{\delta}{(\beta j/N)
+ \delta} \right)\!.
$$

The behavior of $X$ as $n \rightarrow \infty$ is given in the following result, proved in the Supplementary material available at *Biostatistics* online.

Lemma 3 If $r_1 - i_1, \ldots, r_n-i_n \sim Exp(\delta)$ are independent then

$$
\frac{ \sum_{j=1}^{n-1} \sum_{k = j+1}^n ( \omega_{jk} - \mathbb{E}_{{g}}[\omega_{jk}])}{s_n}
= \frac{ \sum_{j=1}^{n-1} \sum_{k = j+1}^n ( \omega_{jk} - \delta^{-1})}{s_n}
$$

converges in distribution to a standard Gaussian random variable as $n \rightarrow \infty$, where

$$\begin{eqnarray*}
s_n^2 & = & \sum_{j=1}^{n-1} \sum_{k = j+1}^n \sum_{l=1}^{n-1} \sum_{m = j+1}^n ( \mathbb{E}_{{g}}[\omega_{jk}\omega_{lm}] - \mathbb{E}_{{g}}[\omega_{jk}] \mathbb{E}_{{g}}[\omega_{lm}])\\
& = & \frac{n(n-1)(2n-1)}{6 \delta^2}.
\end{eqnarray*}$$

Lemma 3 implies that, for large $n$, $X$ is approximately Gaussian with mean ${n \choose 2}\delta^{-1}$ and variance $s_n^2$, and thus the right-hand side of (23) is approximately equal to the moment generating function of this Gaussian distribution evaluated at the point $-\beta/N$. This yields the approximation

$$
\mathbb{E}_{{g}} \left[ \prod_{\substack{j=1\\j \neq \alpha}}^n \psi_j \right] \approx \exp \left\{ - \frac{\beta}{N \delta} {n \choose 2}
+ \frac{\beta^2}{12 \delta^2 N^2}n(n-1)(2n-1) \right\},
$$

which along with (8), (16), and (17) yields a likelihood approximation for large $n$.

#### 3.4.3. Eichner and Dietz approximation with exponential infectious periods

We now consider the ED approximation given in (19) under the assumption that $I_j \sim Exp(\gamma_j)$. First, (20) becomes

$$
\mathbb{E}[\lambda_j(u)] = \sum_{ \substack{k=1\\k \neq j}}^n \beta_{kj} \exp(- \gamma_k(r_k-u))1_{\left\{ u < r_k \right\} },
$$

and direct calculation yields that

$$
\int_{-\infty}^{t} \mathbb{E}[\lambda_j(s)] \; {\rm d}s = \sum_{ \substack{k=1\\k \neq j}}^n \beta_{kj} \gamma_k^{-1} \exp \left\{ - \gamma_k(r_k - (t \wedge r_k)) \right\} = A_j(t),
$$

say. It follows that (19) becomes

$$\begin{eqnarray*}
\pi_{\rm ED}( {r} \,{|}\, {\beta}, {\theta}) & = & \left\{ \prod_{j=1}^n \gamma_j \left( \int_{- \infty}^{r_j}
\sum_{ \substack{k=1\\k \neq j}}^n \beta_{kj} \exp \left\{- \gamma_j (r_j-t) -\gamma_k(r_k -t) -A_j(t) \right\} 1_{\left\{ t < r_k \right\} } \; {\rm d}t \right) \right\}
\nonumber \\
&& \times \exp \left( - \sum_{j=n+1}^N \sum_{k=1}^n \beta_{kj}
\gamma_k^{-1} \right)\!,
\end{eqnarray*}$$

where the integral term does not appear to be available in closed form and thus must be evaluated numerically.

### 3.5. Erlang infectious periods

In this section, we assume that infectious period random variable $I_j$ has an Erlang distribution, i.e. a Gamma distribution with positive integer shape parameter $m_j$ and rate parameter $\nu_j$. Thus ${\theta}_j = (m_j, \nu_j)$, and we write $I_j \sim \Gamma(m_j,\nu_j)$. Such a model is usually more appropriate for real-life diseases than the assumption of exponential infectious period distributions.

Let $x>0$. We have $f_j(x \,{|}\, {\theta}_j) = \nu_j^{m_j} x^{m_j-1} \exp(- \nu_j x)/ (m_j-1)!$ and for $j = 1, \ldots, n$, (7) becomes

$$
g_j(x \,{|}\, {\theta}_j) = \frac{\nu_j^{m_j} x^{m_j-1} \exp(- (\nu_j + B_j) x)/ (m_j-1)!}{[\nu_j/(\nu_j+B_j)]^{m_j}} = \delta_j^{m_j} x^{m_j-1} \exp(- \delta_j x)/ (m_j-1)!,
$$

where $\delta_j = \nu_j + B_j$, so that $g_j$ is the probability density function of a $\Gamma(m_j,\delta_j)$ random variable, and

$$
a({\theta}_j, B_j) = a((m_j,\nu_j), B_j) = [\nu_j/(\nu_j+B_j)]^{m_j} = (\nu_j/\delta_j)^{m_j}.
$$

#### 3.5.1. The standard pair-based approximation with Erlang infectious periods

Lemma 4 Let $1 \leq j,k \leq n$ with $j \neq k$, and $\beta > 0$. Then

$$\begin{eqnarray*}
&&{
\mathbb{E}_{g_j,g_k} \left[ \exp ( - \beta \tau_{kj} ) \right] }\\
&& = \left\{
\begin{array}{ll}
1 + \exp(- \delta_k (r_k - r_j)) \delta_j^{m_j} \sum_{l=0}^{m_k-1} \delta_k^l \left[ \left( \frac{\delta_k}{\delta_k+\beta} \right)^{m_k-l} - 1 \right] & \\
\times \sum_{p=0}^{l} \frac{1}{(l-p)!} {m_j+p-1 \choose p} \frac{(r_k-r_j)^{l-p}}{(\delta_j+\delta_k)^{m_j+p}} & \mbox{if $r_j < r_k$},\\
1 - F_{m_j, \delta_j}(r_j-r_k) \left[ 1 - \left( \frac{\delta_k}{\delta_k+\beta} \right)^{m_k} \right] &\\[6pt]
+ \exp(- \delta_k (r_j - r_k)) \delta_j^{m_j} \sum_{l=0}^{m_k-1} \delta_k^l \left[ \left( \frac{\delta_k}{\delta_k+\beta} \right)^{m_k-l} - 1 \right] & \\
\times \sum_{p=0}^{m_j-1} \frac{1}{(m_j-p-1)!} {l+p \choose p} \frac{(r_j-r_k)^{m_j-p-1}}{(\delta_j+\delta_k)^{l+p+1}} & \mbox{if $r_j > r_k$},\\
\end{array}
\right.
\end{eqnarray*}$$

$$\begin{eqnarray*}
&&{
\mathbb{E}_{g_j,g_k} \left[ 1_{ \left\{ i_k < i_j < r_k \right\} } \exp ( - \beta \tau_{kj} ) \right] }\\
&& = \left\{
\begin{array}{ll}
\exp(- \delta_k (r_k - r_j)) \delta_j^{m_j} \delta_k^{m_k} \sum_{l=0}^{m_k-1} \left( \frac{1}{\delta_k+\beta} \right)^{m_k-l} & \\
\times \sum_{p=0}^{l} \frac{1}{(l-p)!} {m_j+p-1 \choose p} \frac{(r_k-r_j)^{l-p}}{(\delta_j+\delta_k)^{m_j+p}} & \mbox{if $r_j < r_k$},\\
\exp(- \delta_k (r_j - r_k)) \delta_j^{m_j} \delta_k^{m_k} \sum_{l=0}^{m_k-1} \left( \frac{1}{\delta_k+\beta} \right)^{m_k-l} & \\
\times \sum_{p=0}^{m_j-1} \frac{1}{(m_j-p-1)!} {l+p \choose p} \frac{(r_j-r_k)^{m_j-p-1}}{(\delta_j+\delta_k)^{l+p+1}} & \mbox{if $r_j > r_k$},\\
\end{array}
\right.
\end{eqnarray*}$$

where $F_{m, \nu}$ denotes the distribution function of a $\Gamma(m, \nu)$ random variable.

#### 3.5.2. The Eichner and Dietz approximation with Erlang infectious periods

First note that

$$
\mathbb{E}[\lambda_j(u)] = \sum_{ \substack{k=1\\k \neq j}}^n \beta_{kj} 1_{\left\{ u < r_k \right\} } \exp(- \nu_k(r_k-u)) \sum_{l=0}^{m_k-1}
\frac{(\nu_k(r_k-u))^l}{l!},
$$

from which we obtain

$$\begin{eqnarray*}
\int_{-\infty}^{t} \mathbb{E}[\lambda_j(s)] \; {\rm d}s & = & \sum_{ \substack{k=1\\k \neq j}}^n \beta_{kj} \nu_k^{-1} \exp \left\{ - \nu_k(r_k - (t \wedge r_k)) \right\}
\sum_{l=0}^{m_k-1} \frac{ [ \nu_k(r_k - (t \wedge r_k)) ]^l}{l!} (m_k - l) \\
& = & C_j(t),
\end{eqnarray*}$$

say. It follows that

$$\begin{eqnarray*}
&&{\pi_{ED}( {r} \,{|}\, {\beta}, {\theta})=}\\
&&\left\{ \prod_{j=1}^n \left( \int_{- \infty}^{r_j}
f_j(r_j-t \,{|}\, m_j, \nu_j)
\sum_{ \substack{k=1\\k \neq j}}^n \beta_{kj}
\exp \left\{ - \nu_k(r_k -t) - C_j(t) \right\} 1_{\left\{ t < r_k \right\} } \; \sum_{l=0}^{m_k-1} \frac{[\nu_k(r_k-t)]^l}{l!} \; {\rm d}t \right) \right\}
\nonumber \\
&& \times \exp \left( - \sum_{j=n+1}^N \sum_{k=1}^n \beta_{kj} m_k \nu_k^{-1} \right),
\end{eqnarray*}$$

where $f_j(r_j-t \,{|}\, m_j, \nu_j) = \nu_j^{m_j} (r_j-t)^{m_j-1} \exp(- \nu_j (r_j-t))/ (m_j-1)!$.

## 4. Applications to data

Having derived PBLA methods, it is natural to assess their performance for both simulated and real data. Here, we briefly describe the findings of a simulation study, and then illustrate the PBLA methods via three examples involving real-life data. In each case of the latter, the setting goes beyond that of a simple SIR model in a homogeneously mixing population, thus illustrating the potential flexibility of the PBLA methods. For comparison, in each case we also provide results from an alternative analysis such as standard MCMC with data-augmentation.

### 4.1. Simulation study

**Fig. 1.**
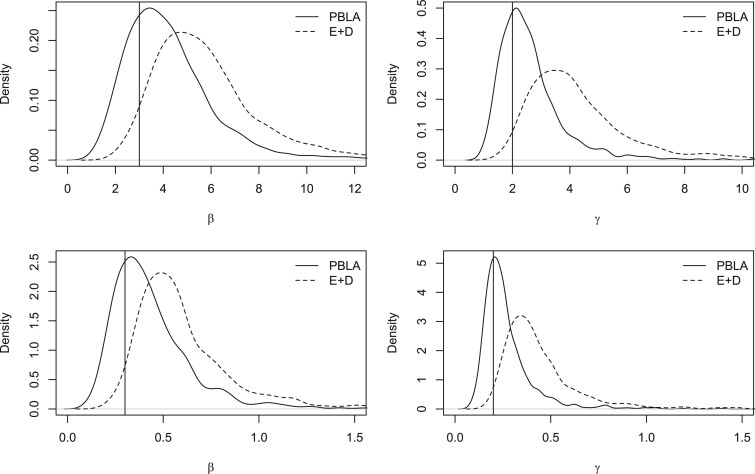
Comparison of maximum likelihood estimates as $\beta$ and $\gamma$ vary, using simulated data sets with exponential infectious periods and $N=100$, $R_0=1.5$. Top panels: $\beta = 3$, $\gamma=2$. Bottom panels: $\beta=0.3$, $\gamma=0.2$. Vertical lines show the true values.

Details of an extensive simulation study can be found in the Supplementary material available at *Biostatistics* online, in which the performance of the PBLA methods is explored for the SIR model across a range of data sets and parameter values. Comparisons with other methods are also described. The focus is on the homogenously mixing case, since it seems natural to assess the methods in this setting. Broadly speaking the methods (i) are found to work well in situations where the proportion of individuals infected is not larger than around 70%, (ii) are competitive with data-augmented MCMC methods for large population sizes, and (iii) improve in accuracy as the shape parameter of the Erlang distribution increases. As an example, Figure 1 shows maximum likelihood estimates taken from 1000 simulated data sets for both the PBLA and the Eichner–Dietz methods. Full details are given in the Supplementary material available at *Biostatistics* online.

### 4.2. Respiratory disease in Tristan da Cunha

We now apply our methods to a data set described in full and analyzed in Becker and Hopper (1983) and Hayakawa *and others* (2003). The data set consists of case diagnosis times of individuals who contracted the common cold during an outbreak which occurred between October and November of 1967 on the island of Tristan da Cunha in the South Atlantic. The population of 255 islanders comprised three age groups, namely infants, children, and adults, which we label 1, 2, and 3, respectively. As there was one unidentified case, we suppose that $N=254$. The initial number of susceptibles in each group are $N_1=25$, $N_2=36,$ and $N_3=192$. We assume that the initial infective is the individual who was diagnosed first, which is not unreasonable since one week elapsed between the first and second diagnosed cases. The number of cases in each group was $n_1 = 9$, $n_2 = 6,$ and $n_2 = 25$.

#### 4.2.1. Transmission model

Following Hayakawa *and others* (2003), we consider a multi-type stochastic SIR model in which the population is divided into three groups. Infectious periods are exponentially distributed with mean $\gamma^{-1}$, and the infection rate from individual $i$ to $j$ is $\beta_{ij} = \beta_{G(j)}$, where $G(j)$ denotes the group (1, 2, or 3) of individual $j$. This model assumes that the population mixes homogeneously and that all infectives are equally infectious, but that the susceptibility of individuals depends on their age group. We relate this model to the data by assuming that case diagnosis times correspond to removal times. We carried out a Bayesian analysis using MCMC in which the target density is the posterior distribution of the four model parameters given the observed removal times under the assumption of the PBLA likelihood. We used the independent prior distributions in Hayakawa *and others* (2003), namely that $\gamma \sim \Gamma(10^{-4}, 10^{-3})$, $\beta_j \sim \Gamma(10^{-8}, 10^{-5})$ for $j=1, 2, 3$.

#### 4.2.2. Results

**Fig. 2.**
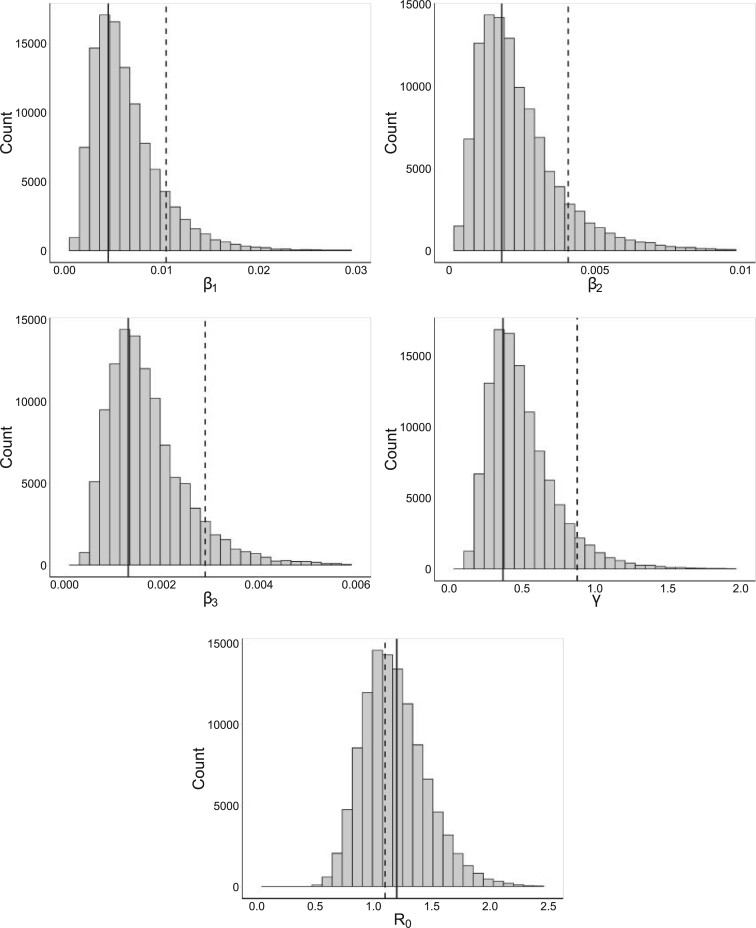
Marginal posterior distributions for the Tristan Da Cunha data using the PBLA method. For comparison, solid lines show the posterior means from Hayakawa *and others* (2003) and dashed lines show maximum *a posteriori* estimates from the Eichner–Dietz method.

Figure 2 shows marginal posterior distributions of the four model parameters from the PBLA analysis and for $R_0$, and also the corresponding posterior means from the analysis in Hayakawa *and others* (2003) which was carried out using MCMC methods featuring data augmentation for the unknown infection times. The latter can be regarded as the “gold-standard” results in the Bayesian setting. Table 1 contains numerical values for the posterior means from the PBLA and data-augmented MCMC approaches. There is good agreement which shows that the PBLA methods provide a good approximation in this case. For comparison, Table 1 and Figure 2 also show maximum *a posteriori* estimates using the Eichner–Dietz approach, which appears to perform rather less well than PBLA here.

**Table 1.** Posterior means from PBLA method and from data-augmented MCMC methods (Hayakawa *and others*, 2003), and maximum *a posteriori* (MAP) estimates using the Eichner–Dietz method, for the Tristan da Cunha data set

	PBLA mean	MCMC mean	E-D MAP
$\beta_1$	0.00641	0.00451	0.0104
$\beta_2$	0.00239	0.00181	0.00408
$\beta_3$	0.00171	0.00121	0.00289
$\gamma$	0.499	0.371	0.879
$R_0$	1.2	1.2	1.1

### 4.3. Ebola in West Africa

Our second example uses publicly available data from the Centers for Disease Control and Prevention (CDC) on the outbreaks of Ebola virus in Guinea, Sierra Leone, and Liberia during 2014. Each data set consists of the numbers of deaths each day due to Ebola. For comparison, we also fit these data to a deterministic epidemic model similar to one proposed by Althaus (2014). This model features latent periods, i.e. it is a Susceptible-Exposed-Infective-Removed (SEIR) model, and also a time-dependent infection rate. We thus have to adapt the PBLA approach to incorporate both these features.

#### 4.3.1. Latent periods in the PBLA framework

The model described in Section 2.1 can be extended to an SEIR model by stipulating that when a susceptible individual $j$ is contacted by an infective, the susceptible immediately enters a latent (or exposed) period at time $e_j$, say, before becoming infective at time $i_j$. During the latent period, the individual is unable to infect others, and cannot themselves be re-infected. In the following, we shall assume that latent periods are of a known fixed duration $c$. Although this is partly for analytical convenience, in reality it is pragmatic to make strong assumptions about either infectious or latent periods if the only available data are removal times. This is essentially because a single data point $r_j$ is insufficient to estimate both $e_j$ and $i_j$ separately without additional assumptions.

Without latent periods, infection times such as $i_j$ play two roles in the PBLA approximation, namely (i) the start of $j$’s infectious period and (ii) the time at which $j$ becomes infected. With latent periods, these times are $i_j$ and $e_j$, respectively. For example, the quantity $\tau_{kj}$ defined at (4) now becomes

$$
\tau_{kj} = r_k \wedge e_j - i_k \wedge e_j .
$$

However, $e_j = i_j - c$, and so the probability distribution of $e_j$ given that $r_j$ is identical to the distribution of $i_j$ given $r_j - c$. By following such arguments, it can be shown the results in Lemmas 1 and 4 can be used for the SEIR model by simply replacing $r_j$ with $r_j - c$ throughout.

#### 4.3.2. Deterministic model for Ebola

Let $s(t)$, $e(t)$, $i(t),$ and $r(t)$ denote respectively the numbers of susceptible, exposed, infective, and removed individuals in a population of size $N$ at time $t$ and assume that $s(t)+e(t)+i(t)+r(t) = N$ for all $t \geq \tau_0$, where $\tau_0$ denotes the initial time point of the epidemic. We define a deterministic model by the set of differential equations

$$\begin{eqnarray*}
\frac{{\rm d}s}{{\rm d}t} & = & - \beta(t) \frac{si}{N},\\
\frac{{\rm d}e}{{\rm d}t} & = & \beta(t) \frac{si}{N} - \sigma e,\\
\frac{{\rm d}i}{{\rm d}t} & = & \sigma e - \gamma i,\\
\frac{{\rm d}r}{{\rm d}t} & = & \gamma i,
\end{eqnarray*}$$

with initial conditions $s(\tau_0) = N-1$, $i(\tau_0)=1$, where

$$
\beta(t) = b_0 \exp (-k(t + \tau_0)).
$$

This model is a simplification of that proposed by Althaus (2014), the difference being that the latter also accounts for non-fatal cases. This means we can compare our methods (which are designed for data on one kind of observation, namely removal times), directly with the deterministic modeling approach without having to make extra assumptions for how to deal with a second kind of observation, namely non-fatal cases.

The time-dependent infection rate $\beta(t)$ is motivated by the impact of control measures; $\tau_0$ is the time at which the initial infective appears and is relevant because when fitting the model to data, it is necessary to decide when the epidemic begins. The parameters $\sigma$ and $\gamma$ are the rates at which individuals move from the exposed to infective and infective to removed classes, respectively.

Following Althaus (2014), we assume that (i) the average lengths of the latent and infectious periods are $\sigma^{-1} = 5.3$ and $\gamma^{-1} = 5.61$ days, respectively, and that $N=10^{6}$ for each country, while (ii) the parameter $\tau_0$ is known for the Guinea outbreak but unknown for the other outbreaks. The remaining parameters $b_0$ and $k$, and $\tau_0$ if required, are estimated from the data as follows, again using the approach of Althaus (2014). First note that the data take the form $\left\{ r(t_i): i = 1, \ldots, M \right\}$, i.e. $M$ observations of the total number of removals, which are assumed to correspond to deaths. A likelihood can be constructed by assuming the observed number of removals at time $t$ is drawn from a Poisson distribution with mean $r(t)$, where $r(t)$ can be computed by numerical solution of the differential equation system, and with independence between different observations. It is then straightforward to obtain numerical maximum likelihood estimates of the model parameters.

#### 4.3.3. PBLA method

To implement the PBLA method, we assume that infectious periods are exponential with mean $\gamma^{-1} = 5.61$ days and latent periods are all $c= \sigma^{-1} = 5.3$ days. As usual, we assume that individuals $1, \ldots, n$ are those who become infected and set $r_1 < \ldots < r_n$. For simplicity, we set $\tau_0$ equal to the estimated values from Althaus (2014). Since the PBLA method assumes that the infection rate between any two individuals is fixed, and not time-dependent, we set

$$
\beta_{jk} = b_0 \exp(-k(T_{jk} + \tau_0)),
$$

where $T_{jk}$ is the expected mid-point of the time during which $j$ can infect $k$. Thus for $1 \leq j,k \leq n$,

$$\begin{eqnarray*}
T_{jk} & = & (\mathbb{E} [ r_j \wedge e_k ] + \mathbb{E} [ i_j \wedge e_k ])/2\\
& = & \left\{
\begin{array}{ll}
r_k - \gamma^{-1} - \sigma^{-1} - (4 \gamma)^{-1} \exp(- \gamma (r_j - r_k + \sigma^{-1})) & \mbox{if $r_j > r_k - \sigma^{-1}$},\\
r_j - (2 \gamma)^{-1} + 3 (4 \gamma)^{-1} \exp ( - \gamma (r_k - r_j - \sigma^{-1})) & \mbox{if $r_j < r_k - \sigma^{-1}$},
\end{array}
\right.
\end{eqnarray*}$$

while for $1 \leq j \leq n$ and $k > n$, $T_{jk} = r_j - (2 \gamma)^{-1}$. We found in practice that other reasonable definitions of $T_{jk}$, e.g. taking into account the exponentially decaying nature of $\beta(t)$, gave similar results.

#### 4.3.4. Results

**Fig. 3.**
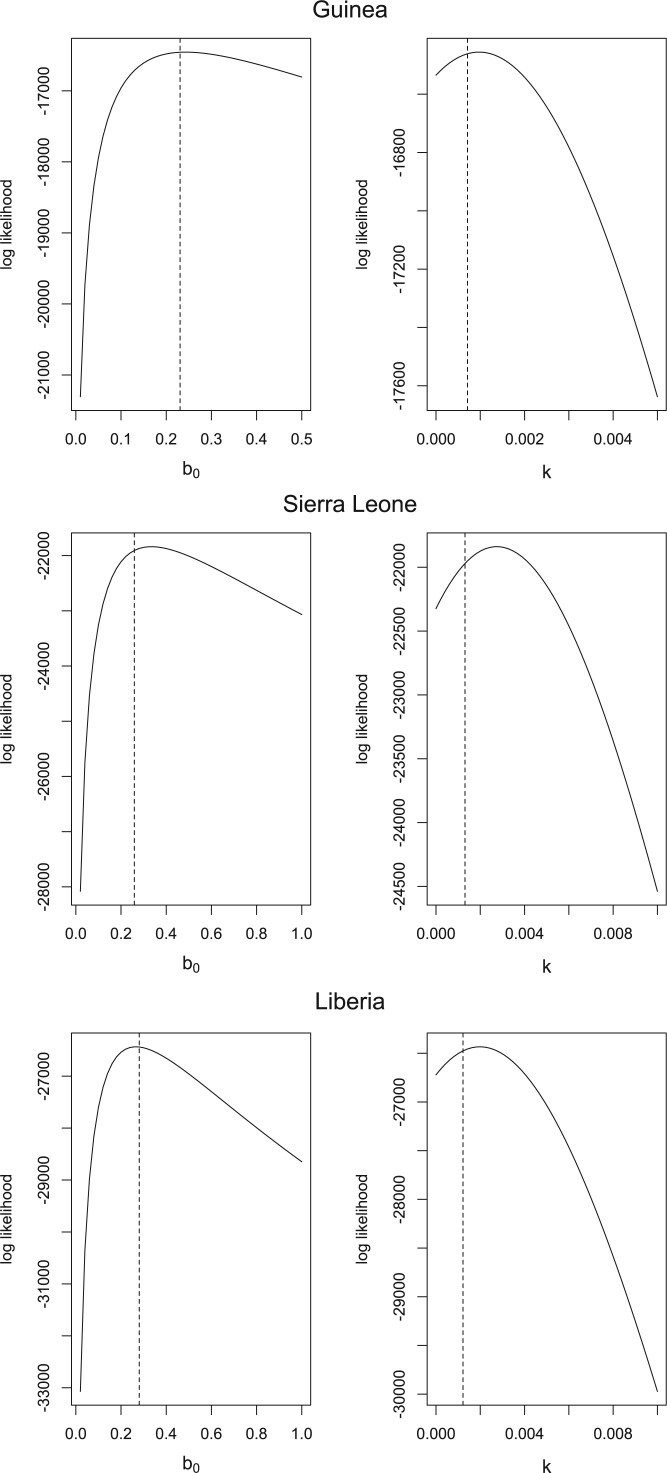
Profile log-likelihoods for $b_0$ and $k$ for the PBLA log-likelihood, using Ebola deaths data from CDC. For comparison, the dashed lines show the maximum likelihood estimates using the Althaus model.

Since the PBLA method is not designed to approximate a Poisson likelihood for an ordinary differential equation model, it is interesting to see how the two approaches compare. Figure 3 shows profile log-likelihood plots for the PBLA method, with maximum likelihood estimates from the Althaus model for comparison. Table 2 contains the numerical values of the maximum likelihood estimates for both approaches. It can be seen that the PBLA method gives reasonably similar results. Point estimates of the basic reproduction number, $R_0 = \beta_0 / \gamma$, for PBLA (Althaus) are 1.4 (1.3), 1.9 (1.6), and 1.7 (1.5) for Guinea, Sierra Leone, and Liberia, respectively, which again show reasonable agreement.

**Table 2.** Maximium likelihood estimates from the PBLA method and from the Althaus model for the Ebola deaths data from CDC

		$b_0$	$k$
	Guinea	0.243	0.00105
PBLA	Sierra Leone	0.335	0.00289
	Liberia	0.266	0.00214
	Guinea	0.231	0.000712
Althaus	Sierra Leone	0.277	0.00180
	Liberia	0.303	0.00251

### 4.4. Foot-and-Mouth disease in Cumbria, UK

Our final application concerns a large data set taken from the 2001 Foot and Mouth disease outbreak in the UK county of Cumbria. In this outbreak, the disease spread between farms, and if detected on a farm the animals there were culled in order to prevent further transmission. The particular data that we consider are described in Kypraios (2007), Jewell *and others* (2009), and Xiang and Neal (2014). In summary, for each farm in Cumbria the data comprise (i) its geographic location, (ii) the numbers of cattle and sheep, and (iii) the culling date if the farm was deemed to have been infected. In total, $n=1021$ of $N=5378$ farms were infected.

#### 4.4.1. Transmission model

Kypraios (2007) describes a stochastic SIR transmission model in which individuals are farms, infectious periods are assumed to follow independent $\Gamma(4, \gamma)$ distributions, and the infection rate from farm $i$ to farm $j$ is given by

$$
\beta_{ij} = \beta_0 \frac{v}{\rho_{ij}^2 + v^2} ( \varepsilon (n^c_i)^\zeta + (n^s_i)^\zeta)( \xi (n^c_j)^\zeta + (n^s_j)^\zeta),
$$

where $\rho_{ij}$ denotes the Euclidean distance between farms $i$ and $j$, while $n^c_i$ and $n^s_i$ denote respectively the number of cattle and sheep on farm $i$. The model is thus explicitly spatial and multi-type, and has six parameters. This model is related to the data by assuming that culling dates correspond to removal events. Kypraios (2007) carries out parameter estimation in a Bayesian framework by augmenting the parameter space with the unknown infection times. This is a very computationally demanding approach, due to the combination of a large number of infected farms, a six-dimensional model parameter space, and the inherent posterior correlations between the infection times and the model parameters.

We adopted the same independent prior distributions as those of Kypraios (2007), namely that $\beta_0, \gamma \sim \Gamma(0.001, 0.001)$, $v \sim {\rm Exp}(0.1),$ and $\varepsilon, \xi, \zeta \sim {\rm Exp}(0.001)$. We used the PBLA method to obtain maximum *a posteriori* (MAP) point estimates for all six parameters.

#### 4.4.2. Results

**Fig. 4.**
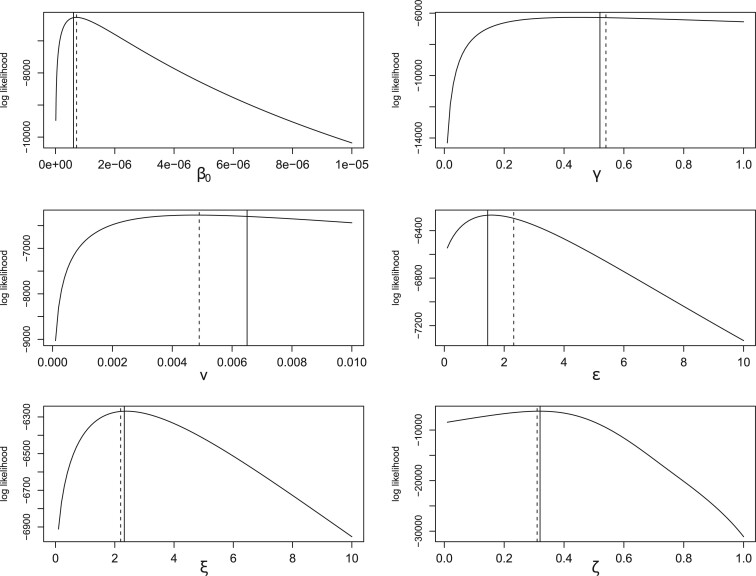
Profile log-likelihoods for the Foot and Mouth disease model parameters. For comparison, the solid lines show posterior mean estimates from Kypraios (2007) and the dashed lines show corresponding Eichner–Dietz MAP estimates.

Figure 4 shows profile log-likelihood plots along with the posterior mean estimates from Kypraios (2007), and Table 3 compares the latter with the MAP estimates from the PBLA analysis. MAP estimates from the Kypraios analysis are not available, but since the marginal posterior density plots reported are reasonably symmetric then the posterior means would presumably be fairly close. It can be seen that there is reasonable agreement between the PBLA approach and the Kypraios analysis. For comparison, we also present the corresponding MAP estimates from the Eichner–Dietz approximation, which are slightly less accurate than the PBLA estimates.

**Table 3.** MAP estimates (PBLA method) and posterior means from Kypraios (2007) for the Foot and Mouth data set, along with the relative difference $|\hat{{\theta}}_{\rm MAP} - E[{\theta} \,{|}\, {r} ]|/E[{\theta} \,{|}\, {r} ]$. For comparison, the final column shows the Eichner–Dietz MAP estimates

${\theta}$	$\hat{{\theta}}_{\rm MAP}$ (PBLA)	$E[{\theta} \,{|}\, {r} ]$	Rel. Diff.	$\hat{{\theta}}_{\rm MAP}$ (E-D)
$\beta_0$	$7.05 \times 10^{-7}$	$6.07 \times 10^{-7}$	0.16	$7.10 \times 10^{-7}$
$\gamma$	0.45	0.52	0.13	0.54
$v$	0.0048	0.0065	0.26	0.0049
$\varepsilon$	1.57	1.45	0.08	2.32
$\xi$	2.39	2.32	0.03	2.20
$\zeta$	0.32	0.32	0	0.31

## 5. Conclusions

We have developed likelihood approximation methods for partially observed stochastic epidemic models. We regard such methods as an addition to the toolkit for analyzing infectious disease data, with potential to provide parameter estimates in situations where other methods (such as the “gold-standard” of data-augmented MCMC, or likelihood-free methods such as Approximate Bayesian Computation) may struggle due to their computational burden. Our work is somewhat preliminary but demonstrates that likelihood-approximation approaches have useful potential.

Our approach can be summarized as follows. Broadly speaking, the true likelihood can be described by considering all individuals who ever become infected. It then takes account of these individuals avoiding infection, becoming infected, remaining infected for a period of time, and either infecting or failing to infect others whilst infective. Due to the fact that these events are dependent for different individuals, the likelihood is then typically intractable. The key to our approximation method is to consider pairs of ever-infected individuals in the population, since the likelihood contribution from such pairs are often tractable, depending on the choice of infectious period distribution. We then assume independence between such pairs in order to form an approximate likelihood.

The PBLA approach appears to work reasonably well in settings encountered in practice, specifically where the total proportion of the population infected is not too large. This makes intuitive sense, since the extent to which the likelihood can be approximated by independent components will clearly become less plausible as the proportion infected increases. The general idea of basing approximations on the interactions of pairs of individuals is widely applicable, as we have demonstrated via several examples. It seems likely that such methods could also be applied to models where individuals enter and leave the population, for instance models for nosocomial infections in hospital wards (Wei *and others*, 2018). Another possible extension is to epidemics in progress, relaxing our assumption that the observed outbreak has terminated. The challenge in that situation is that both the infection times and total number of ever-infected individuals in the population are both unknown.

Unlike data-augmented MCMC, the PBLA approach gives a relatively fast way to obtain approximate maximum likelihood values for model parameters. For small data sets, the PBLA method can produce maximum likelihood estimates in seconds or less, while even the Ebola data set with $N=10^6$ individuals takes around 1 min on a standard laptop. Further work is needed to develop approaches that can improve the speed and accuracy of the approximation across more scenarios, for instance by taking greater account of the fact that not all configurations of the unknown infection times are possible if the epidemic is not to die out prematurely.

## Supplementary Material

kxz053_Supplementary_Data

## Supplementary material

Supplementary material is available online at http://biostatistics.oxfordjournals.org and code and data sets from the paper are available at https://github.com/jessicastockdale/PBLA.
