# Supplementary material for: Pair-based likelihood approximations for stochastic epidemic models
Source: Biostatistics. 2019 Dec 6;22(3):575–97. doi: 10.1093/biostatistics/kxz053 (PMC8286555; doi:10.1093/biostatistics/kxz053)
Supplement: kxz053_Supplementary_Data [file kxz053_supplementary_data.pdf]

Pair-based likelihood approximations for stochastic epidemic models Supplementary Material

J. E. Stockdale[*], T. Kypraios[†], P. D. O'Neill[†]

1 Proofs

1.1 Proofs of Lemmas 1 and 4

We give two methods of proof for the first result in lemma 1; the other parts of both lemmas can be proved in a similar fashion. First note that

$$\tau_{kj} = r_k \wedge i_j - i_k \wedge i_j = \begin{cases} 0 & \text{if } i_j < i_k, \\ i_j - i_k & \text{if } i_k < i_j < r_k, \\ r_k - i_k & \text{if } i_j > r_k. \end{cases}$$

Thus

$$\begin{aligned} &\mathbb{E}_{g_j,g_k}\left[\exp(-\beta\tau_{kj})\right] \\ &\quad = \mathbb{E}_{g_j,g_k}\left[\exp(-\beta\tau_{kj})1_{\{i_j<i_k\}}\right] + \mathbb{E}_{g_j,g_k}\left[\exp(-\beta\tau_{kj})1_{\{i_k<i_j<r_k\}}\right] + \mathbb{E}_{g_j,g_k}\left[\exp(-\beta\tau_{kj})1_{\{i_j>r_k\}}\right] \\ &\quad = \mathbb{E}_{g_j,g_k}\left[1_{\{i_j<i_k\}}\right] + \mathbb{E}_{g_j,g_k}\left[\exp(-\beta(i_j-i_k))1_{\{i_k<i_j<r_k\}}\right] + \mathbb{E}_{g_j,g_k}\left[\exp(-\beta(r_k-i_k))1_{\{i_j>r_k\}}\right] \end{aligned} \quad (1)$$

and all three terms in (1) can be evaluated directly; for instance, if $r_j < r_k$ then

$$\begin{aligned} \mathbb{E}_{g_j,g_k}\left[1_{\{i_j<i_k\}}\right] &= \int_{-\infty}^{r_j}\int_{i_j}^{r_k} \delta_k \exp(-\delta_k(r_k-i_k))\delta_j \exp(-\delta_j(r_j-i_j))\, di_k di_j \\ &= 1-\delta_j(\delta_j+\delta_k)^{-1}\exp(-\delta_k(r_k-r_j)), \end{aligned} \quad (2)$$

$$\begin{aligned} &\mathbb{E}_{g_j,g_k}\left[\exp(-\beta(i_j-i_k))1_{\{i_k<i_j<r_k\}}\right] \\ &\quad = \int_{-\infty}^{r_j}\int_{-\infty}^{i_j} \exp(-\beta(i_j-i_k))\delta_k\exp(-\delta_k(r_k-i_k))\delta_j\exp(-\delta_j(r_j-i_j))\, di_k di_j \\ &\quad = \delta_j\delta_k\left\{(\delta_j+\delta_k)(\delta_k+\beta)\right\}^{-1}\exp(-\delta_k(r_k-r_j)), \end{aligned} \quad (3)$$

[*]Department of Mathematics, Simon Fraser University
[†]School of Mathematical Sciences, University of Nottingham

and the third term is zero since $i_j < r_j < r_k$, whence $1_{\{i_j > r_k\}} = 0$. Combining (2) and (3) yields the first result in lemma 1.

Alternatively, a direct probabilistic proof is as follows. From (1) we have

$$\begin{aligned}&\mathbb{E}_{g_j,g_k}\left[\exp(-\beta\tau_{kj})\right]\\&\quad= \mathbb{P}_{g_j,g_k}(i_j < i_k) + \mathbb{E}_{g_j,g_k}\left[\exp(-\beta(i_j - i_k))|i_k < i_j < r_k\right]\mathbb{P}_{g_j,g_k}(i_k < i_j < r_k).\end{aligned} \tag{4}$$

Both terms in (4) can be evaluated by considering a reverse-time construction of i_j and i_k, as follows (see also the proof of lemma 2 below for a similar approach). Recall that $r_j < r_k$. Start at time r_k and run a Poisson process of rate δ_k backwards in time. If a point occurs at time $t \in (r_j, r_k)$ then set $i_k = t$, and run a new Poisson process of rate δ_j backwards in time from r_j and set i_j equal to the time of its first point. If not, at time r_j increase the Poisson process rate to $\delta_j + \delta_k$; when the first point appears at time $t < r_j$, set $i_j = t$ with probability $\delta_j(\delta_j + \delta_k)^{-1}$, otherwise set $i_k = t$. In the former case reduce the Poisson process rate to δ_k, and in the latter reduce it to δ_j. Continue back in time; the next point to appear will be the remaining infection time. It is straightforward to see that this construction yields i_j and i_k such that $r_j - i_j$ and $r_k - i_k$ are independent $Exp(\delta_j)$ and $Exp(\delta_k)$ random variables, respectively.

Under this construction, $i_k < i_j$ if and only if (i) the Poisson process has no point in (r_j, r_k), which has probability $\exp(-\delta_k(r_k - r_j))$, and (ii) independently of this, i_j is chosen when the first point of the rate-$(\delta_j + \delta_k)$ Poisson process appears, which has probability $\delta_j(\delta_j + \delta_k)^{-1}$. Thus the first term in (4) is

$$\mathbb{P}_{g_j,g_k}(i_j < i_k) = 1 - \mathbb{P}_{g_j,g_k}(i_k < i_j) = 1 - \delta_j(\delta_j + \delta_k)^{-1}\exp(-\delta_k(r_k - r_j)),$$

in agreement with (2). Next, since $i_j < r_j < r_k$ we have

$$\mathbb{P}_{g_j,g_k}(i_k < i_j < r_k) = \mathbb{P}_{g_j,g_k}(i_k < i_j) = \delta_j(\delta_j + \delta_k)^{-1}\exp(-\delta_k(r_k - r_j)). \tag{5}$$

Finally, to evaluate the expectation term in (4), consider a Poisson process of rate β that runs backwards in time from i_j, independently of the rate-δ_k Poisson process that is used if $i_k < i_j$. The expectation is simply the probability that the former process has no points before the first point of the latter process, yielding

$$\mathbb{E}_{g_j,g_k}\left[\exp(-\beta(i_j - i_k))|i_k < i_j < r_k\right] = \delta_k(\delta_k + \beta)^{-1}$$

which along with (5) yields the same expression as (3).

The remaining cases in lemma 1, and 4, can be proved using either direct calculation or probabilistic arguments. For the case of Erlang distributed infectious periods, the probabilistic arguments require splitting infectious periods into exponentially-distributed stages. Full details can be found in Stockdale (2018).

1.2 Proof of Lemma 2

Note that in the following, we focus exclusively on the individuals in $\mathcal{K}$ and ignore all other individuals in the population. For ease of exposition, re-label the members of $\mathcal{K}$ as $1, \ldots, K$. Given these individuals' removal times $r_1 < \ldots < r_K$, their infection times $i_1, \ldots, i_K$ can be constructed as follows. Start at time r_K and consider the Markov process $\left\{(\tilde{S}(t), \tilde{I}(t)) : t < r_K\right\}$ which runs backwards in time such that (i) $(\tilde{S}(t), \tilde{I}(t)) \to (\tilde{S}(t) + 1, \tilde{I}(t) - 1)$ at rate $\delta\tilde{I}(t)$; (ii) at each removal time, $\tilde{I}(t)$ increases by 1. Here $\tilde{S}(t)$ and $\tilde{I}(t)$ denote respectively the numbers of susceptibles and infectives in $\mathcal{K}$ at time t and initially (i.e. just before time r_K) there are no susceptibles and one

infective. Transitions of type 1 generate infection times, and at each such event one of the currently infected individuals is selected uniformly at random to be the individual who is infected at that time. The process terminates as soon as $S(t) = K$, which occurs when the last of the required infection times is generated; call this time i_a.

Consider the total time during which infectious pressure is exerted, i.e.

$$\sum_{j<k} \omega_{jk} = \int_{i_a}^{r_K} \tilde{S}(t)\tilde{I}(t)\, dt.$$

This quantity can be similarly constructed in reverse time as follows. For $u < r_K$ define

$$T(u) = \int_{u}^{r_K} \tilde{S}(t)\tilde{I}(t)\, dt$$

and observe that, starting at r_K and moving backwards in time, T is a piecewise linear function that increases at rate $\tilde{S}(t)\tilde{I}(t)$ at time t.

Next, consider a random time-transformation in which (i) the clock runs at rate $\tilde{I}^{-1}$ when there are $\tilde{I} \geq 1$ infectives, and (ii) the clock stops running whenever $\tilde{I} = 0$. This has no impact on the ultimate value of T, because in each time period during which $\tilde{S}(t)\tilde{I}(t)$ does not change, T increases by the same amount in both the original and transformed time scales. In the transformed time scale, still in reverse time, (i) T increases at rate $\tilde{S}$ when there are $\tilde{S}$ susceptibles, and removal times do not affect this rate because they do not change the number of susceptibles, while (ii) the number of susceptibles increases by one at rate δ, corresponding to an increase at rate $\delta\tilde{I}(t)$ in the original time-scale.

Combining these observations means that, in the transformed time-scale, T is as a function which starts at zero, and as soon as the first susceptible appears increases at rate 1; then increases at rate 2 once the second susceptible appears, and so on until all K susceptibles appear. Since the times at which susceptibles appear are the points of a Poisson process of rate δ, it follows that

$$\sum_{j<k} \omega_{jk} = T(i_a) = \sum_{j=1}^{K-1} jY_j$$

where the Y_j are independent $Exp(\delta)$ random variables, and the result follows since $jExp(\delta) \sim Exp(\delta/j)$.

1.3 Proof of Lemma 3

1.3.1 Preliminaries

For $n \geq 1$ let $D_n = \{(i,j) : 1 \leq i < j \leq n\}$ denote the set of 2-element subsets of $\mathcal{N}_n = \{1, \ldots, n\}$. For $(i,j), (k,l) \in D_n$, $|(i,j) \cap (k,l)|$ is the number of elements of $\mathcal{N}_n$ that (i,j) and (k,l) have in common, which can equal either 0, 1 or 2. We require the following result, which is essentially Theorem 2.1 from Barbour and Eagleson (1985) restricted to our setting.

Lemma 1 *Let* $\{X_{ij} : (i,j) \in D_n\}$ *be a collection of zero-mean random variables such that* $E[|X_{ij}|^3] < \infty$ *for all* $(i,j) \in D_n$*, and* X_{ij} *and* X_{kl} *are independent if* $|(i,j) \cap (k,l)| = 0$*. Let*

$$\sigma_n^2 = \sum_{(i,j),(k,l) \in D_n} E[X_{ij}X_{kl}].$$

Then $\sigma_n^{-1}\sum_{(i,j)\in D_n} X_{ij}$ *converges in distribution as* $n\to\infty$ *to a standard normal random variable if*

$$\sigma_n^{-3}\sum_{(i,j)\in D_n}(E[|X_{ij}|^3])^{1/3}\left(\sum_{(k,l):|(i,j)\cap(k,l)|=0}(E[|X_{kl}|^3])^{1/3}\right)^2\to 0. \quad (6)$$

1.3.2 Proof of Lemma 3

For $(i,j)\in D_n$ set $X_{ij}=\omega_{ij}-E[\omega_{ij}]=\omega_{ij}-\delta^{-1}$, since $\omega_{ij}\sim Exp(\delta)$ from lemma 2. Then $E[X_{ij}]=0$, $E[X_{ij}^2]=\delta^{-2}$, $(E[|X_{ij}|^3])^{1/3}=c>0$, say, $E[X_{ij}X_{kl}]=E[\omega_{ij}\omega_{kl}]$ and $\sigma_n^2=s_n^2$. Thus the left hand side of (6) equals $\sigma_n^{-3}c^3\binom{n}{2}\binom{n-2}{2}^2$ and the result follows if $\sigma_n^3=O(n^{4+\eta})$ for some $\eta>0$. Now

$$\begin{aligned}
\sigma_n^2 &= \sum_{(i,j),(k,l)\in D_n} E[X_{ij}X_{kl}]\\
&= \sum_{\substack{(i,j),(k,l)\in D_n\\ |(i,j)\cap(k,l)|=0}} E[X_{ij}X_{kl}] + \sum_{\substack{(i,j),(k,l)\in D_n\\ |(i,j)\cap(k,l)|=1}} E[X_{ij}X_{kl}] + \sum_{\substack{(i,j),(k,l)\in D_n\\ |(i,j)\cap(k,l)|=2}} E[X_{ij}X_{kl}]\\
&= \sum_{\substack{(i,j),(k,l)\in D_n\\ |(i,j)\cap(k,l)|=0}} E[X_{ij}]E[X_{kl}] + \sum_{\substack{(i,j),(k,l)\in D_n\\ |(i,j)\cap(k,l)|=1}} E[X_{ij}X_{kl}] + \sum_{(i,j)\in D_n} E[X_{ij}^2]\\
&= \sum_{\substack{(i,j),(k,l)\in D_n\\ |(i,j)\cap(k,l)|=1}} E[X_{ij}X_{kl}] + \binom{n}{2}\delta^{-2}.
\end{aligned}$$

For $1\le j<k<l\le n$ define

$$\Omega_{jkl}=\omega_{jk}\omega_{jl}+\omega_{jk}\omega_{kl}+\omega_{jl}\omega_{kl},$$

whence

$$\sum_{\substack{(i,j),(k,l)\in D_n\\ |(i,j)\cap(k,l)|=1}} E[X_{ij}X_{kl}] = \sum_{1\le j<k<l\le n} E[\Omega_{jkl}].$$

Now

$$\begin{aligned}
E[\Omega_{jkl}] &= \mathrm{cov}(\omega_{jk},\omega_{jl})+\mathrm{cov}(\omega_{jk},\omega_{kl})+\mathrm{cov}(\omega_{jl},\omega_{kl})+E[\omega_{jk}]E[\omega_{jl}]+E[\omega_{jk}]E[\omega_{kl}]+E[\omega_{jl}]E[\omega_{kl}]\\
&= \mathrm{cov}(\omega_{jk},\omega_{jl})+\mathrm{cov}(\omega_{jk},\omega_{kl})+\mathrm{cov}(\omega_{jl},\omega_{kl})+3\delta^{-2}\\
&= \left(\mathrm{var}(\omega_{jk}+\omega_{jl}+\omega_{kl})-\mathrm{var}(\omega_{jk})-\mathrm{var}(\omega_{jl})-\mathrm{var}(\omega_{kl})\right)/2+3\delta^{-2}\\
&= \left(5\delta^{-2}-3\delta^{2})\right)/2+3\delta^{-2}\\
&= 4\delta^{-2},
\end{aligned}$$

since $\mathrm{var}(\omega_{jk}+\omega_{jl}+\omega_{kl})=\mathrm{var}(Exp(\delta))+\mathrm{var}(Exp(\delta/2))=5\delta^{-2}$ from lemma 2 with $K=3$. Thus

$$\sigma_n^2=4\binom{n}{3}\delta^{-2}+\binom{n}{2}\delta^{-2}=\frac{n(n-1)(2n-1)}{6\delta^2}=O(n^3),$$

and so $\sigma_n^3=O(n^{9/2})$ as required.

2 Simulation study

In this section we assess the performance of the PBLA and ED approximations using simulated data. Throughout we assume that $\beta_{ij} = \beta N^{-1}$ for all i, j, and that infectious periods are identically distributed, since it seems most natural to assess the methods in the most basic setting. More complex settings are considered in the real-life data examples in the main text. We will also be concerned with the basic reproduction number R_0, defined as the number of infections caused by a single infective in an infinite population of susceptibles (see e.g. Andersson and Britton, 2000).

Our main focus is on the standard PBLA method, since we found that the alternative approximations described in section 3.3 in the main text were either numerically similar, or performed less well (see 2.4 below for details).

2.1 Exponential infectious periods

Suppose that $I_j \sim Exp(\gamma)$ for all $j = 1, \ldots, n$. Then $R_0 = \beta\gamma^{-1}$.

We first consider the performance of the PBLA and ED methods as various combinations of β, γ, R_0 and N vary. Specifically, in each setting we simulate a large number of data sets, typically 500 or 1000, each consisting of removal times $r_1, \ldots, r_n$. For each data set we then find maximum likelihood estimates of (β, γ) for both the PBLA and ED approximation using standard numerical optimisation methods. To provide a visual representation of the estimates we use them to create kernel density estimates.

2.1.1 Varying β and γ with N and R_0 fixed

Figure 1 illustrates that both the PBLA and ED methods are largely unaffected by variations in the actual values of β and γ, at least while N and R_0 are fixed. The PBLA method appears to perform slightly better in terms of being able to estimate the true parameter values.

2.1.2 Varying N with β and γ fixed

Figure 2 shows the impact of varying N while β and γ are fixed. Again we see that there is little variation, and that PBLA performs better than the ED method. Note that since R_0 is fixed, increasing N leads to larger values of the outbreak size n, and so here we see evidence that both approximation methods are able to cope with large n values.

2.1.3 Varying R_0

Figure 3, in conjunction with the top panel in Figure 2, shows how variation in R_0 impacts the performance of the PBLA and ED methods, specifically for $R_0 = 0.5, 1.5$ and 2. For both approximations, the performance deteriorates as R_0 increases. To understand this, first note that the value of $N - n$ contributes to the true likelihood (5), in this case via the product term

$$\prod_{j=1}^{n} \phi_j = \exp\left\{ -\beta N^{-1}(N-n) \sum_{j=1}^{n} (r_j - i_j) \right\}.$$

As the proportion of the population infected, n/N, increases, so the relative importance of the ϕ terms compared to the other terms in (5) decreases, and thus to obtain a good overall approximation it becomes increasingly important to have a good approximation for the ψ terms. However, the latter are themselves the subject of the least accurate approximation, essentially because each of

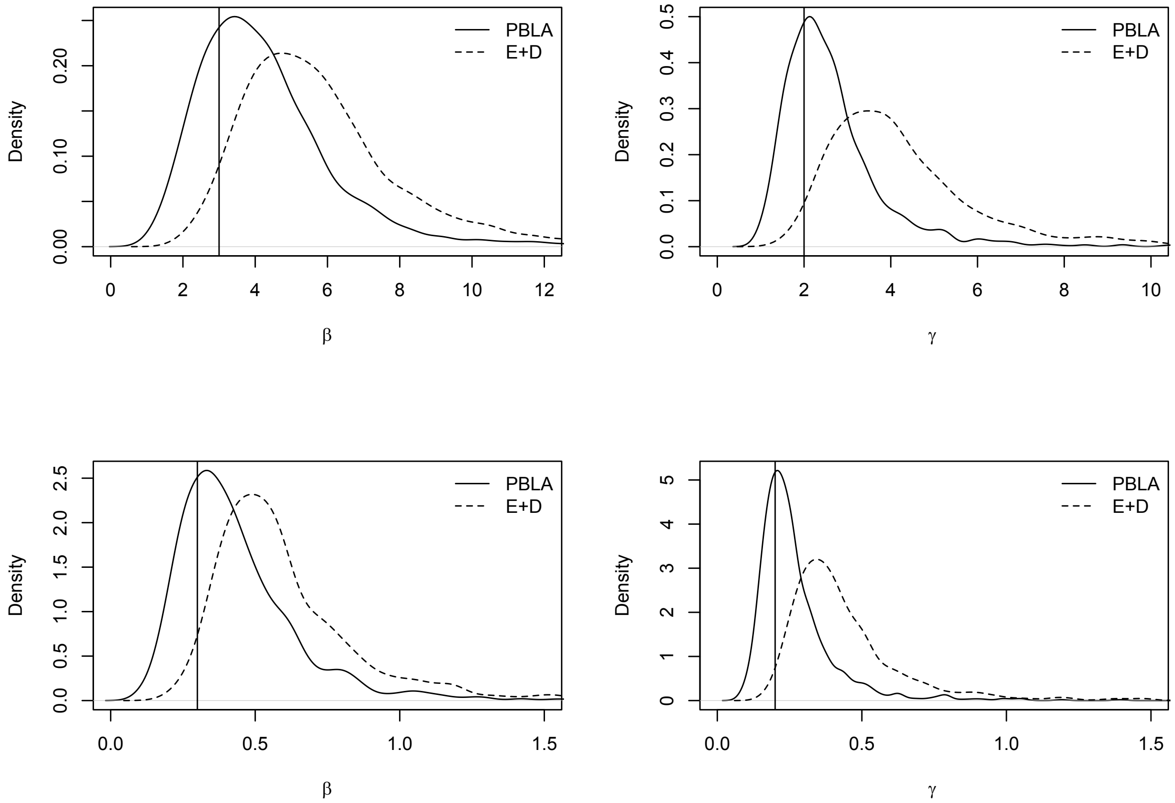

Figure 1: Comparison of maximum likelihood estimates as β and γ vary, using simulated data sets with exponential infectious periods and $N = 100$, $R_0 = 1.5$. Top panels: $\beta = 3$, $\gamma = 2$. Bottom panels: $\beta = 0.3$, $\gamma = 0.2$. Vertical lines show the true values.

the ψ_j terms is itself approximated by a product. Roughly speaking, we found that provided n/N is less than around 0.7, then the relative importance of the ϕ terms is sufficient to provide good approximations for β and γ. However, for larger values of n/N this is no longer the case, and the overall approximation suffers. From a practical point of view this does not seem to be particularly restrictive, since real-life outbreaks rarely infect such high proportions of the susceptible population.

2.2 Erlang infectious periods

Suppose now that $I_j \sim \Gamma(m, \gamma)$ for all $j = 1, \ldots, n$, where m is a positive integer. Then $R_0 = \beta m \gamma^{-1}$. We now briefly show that the broad conclusions of the simulation study for the case of exponential infectious periods also hold true in this setting. We also show that the approximations improve as the variance of the infectious period decreases while its mean is kept fixed.

2.2.1 Varying β and γ with N and R_0 fixed

Figure 4 illustrates that both the PBLA and ED methods are largely unaffected by variations in the actual values of β and γ, at least while N and R_0 are fixed. The PBLA method appears to perform slightly better in terms of being able to estimate the true parameter values.

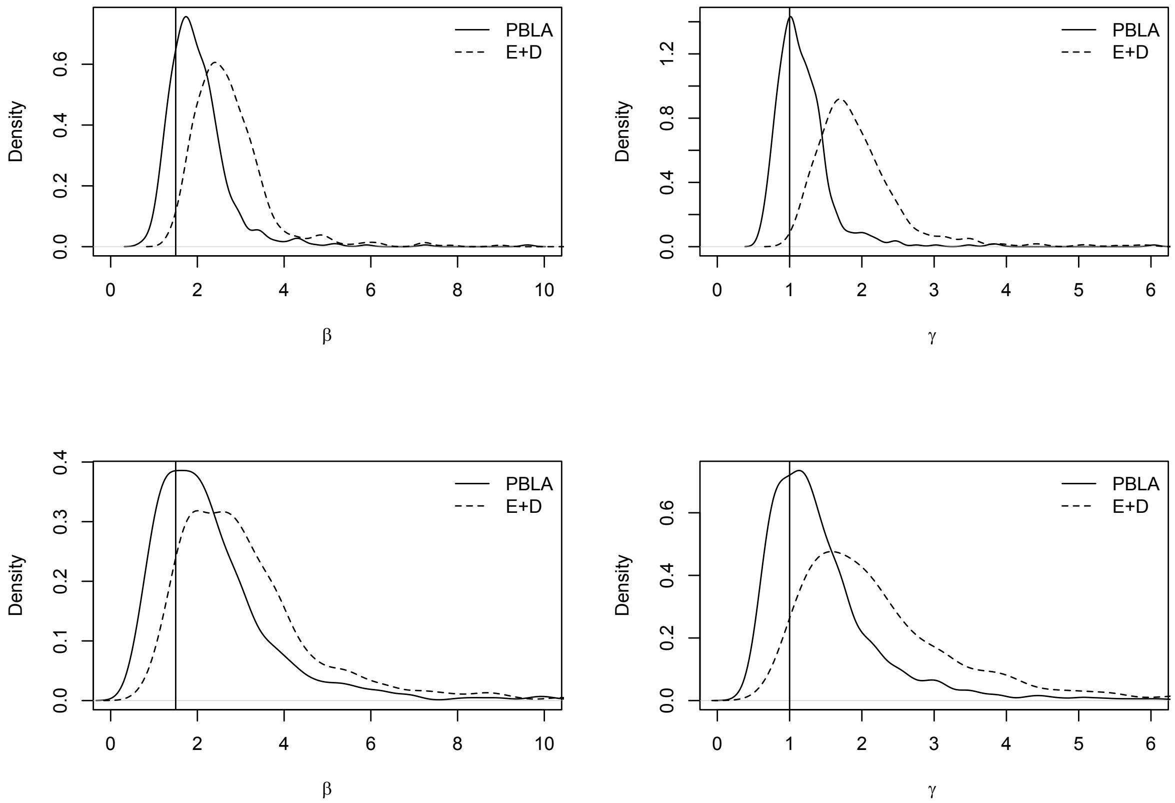

Figure 2: Comparison of maximum likelihood estimates as N varies, using simulated data sets with exponential infectious periods and $\beta = 1.5$, $\gamma = 1$, $R_0 = 1.5$. Top panels: $N = 500$. Bottom panels: $N = 40$. Vertical lines show the true values.

2.2.2 Varying N with β and γ fixed

Figure 5 shows the impact of varying N while β and γ are fixed. The results are broadly similar to the exponential infectious period case, with relatively little variation in the mode of the estimates as N increases, but that the variance of the estimates decreases with N. The latter feature suggests that having more data enables better estimation in the sense that the average error is reduced. We illustrate this graphically in figure 6 which shows mean square error values as N varies. This error reduction is clearly a desirable property of both the PBLA and ED approximation methods, but one that is not obviously true at first sight since larger data sets lead to more approximations being used in both methods.

2.2.3 Varying R_0 with m and γ fixed.

Figure 7 shows the impact of varying R_0 whilst keeping the infectious period distribution parameters m and γ fixed. As for the exponential infectious periods case, and for the same underlying reasons, estimation for both β and γ becomes less accurate as R_0 increases.

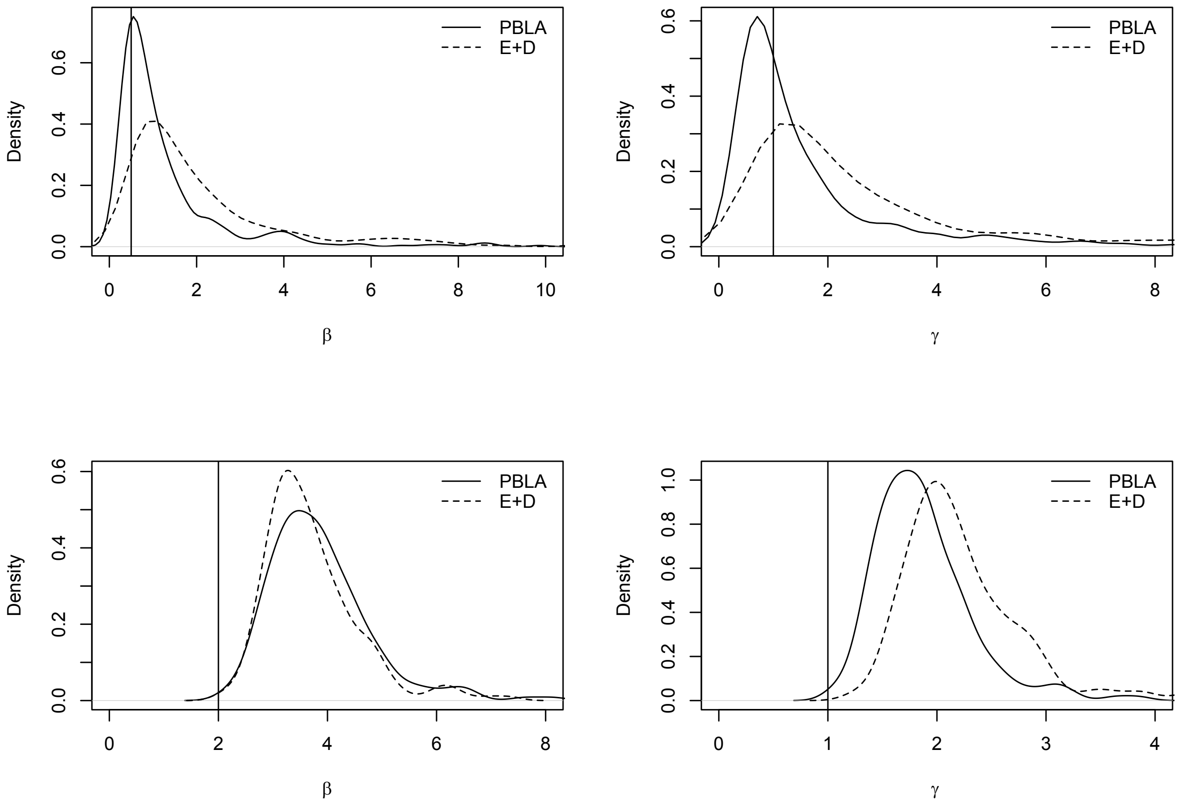

Figure 3: Comparison of maximum likelihood estimates as R_0 varies, using simulated data sets with exponential infectious periods and $N = 500$. Top panels: $\beta = 0.5$, $\gamma = 1$, $R_0 = 1.5$. Bottom panels: $\beta = 2$, $\gamma = 1$, $R_0 = 2$. Vertical lines show the true values.

2.2.4 Varying m with N and R_0 fixed.

If the mean of the infectious period distribution, m/γ, is kept fixed, then increasing m will reduce the variability of the infectious period. In this situation we expect the accuracy of both the ED and PBLA methods to improve, since the underlying assumptions of independence between different components of the likelihood become less unrealistic. Figures 8 and 9 illustrate that this is indeed the case. What is most striking is that marked improvement in estimation from $m = 1$ and $m = 2$, while increasing m further only provides modest further gains.

2.3 Computational performance

One motivation for the PBLA approach is to dispense with the need for data augmentation. However, since the PBLA likelihood can be costly to compute, it is natural to ask how it compares to the standard data-augmented MCMC (DA-MCMC) method described in O'Neill and Roberts (1999) which generates samples from the posterior density $\pi(\beta, \gamma | \boldsymbol{r})$. To address this question we first generated several simulated data sets, consisting of removal times, from the SIR model. On each data set we then ran both the standard DA-MCMC algorithm, with β, γ and all unknown infection times updated at each iteration, and an MCMC algorithm with the PBLA likelihood in which β and

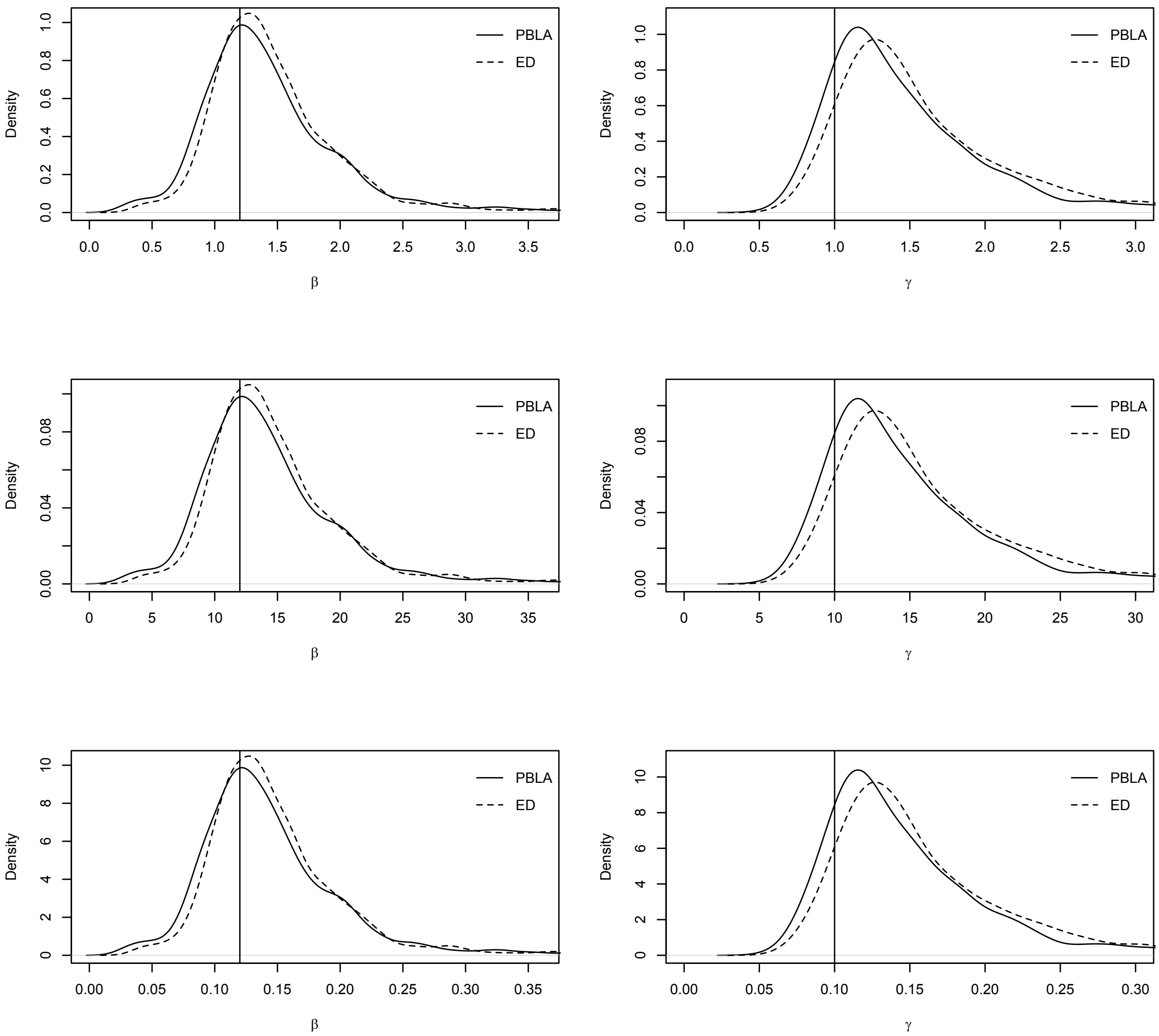

Figure 4: Comparison of maximum likelihood estimates as β and γ vary, using simulated data sets with Erlang infectious periods and $N = 100$, $m = 2$, $R_0 = 2.4$. Top panels: $\beta = 12$, $\gamma = 10$. Middle panels: $\beta = 1.2$, $\gamma = 1$. Bottom panels: $\beta = 0.12$, $\gamma = 0.1$. Vertical lines show the true values.

γ were updated separately at each iteration using a Gaussian random walk proposal mechanism. Both algorithms were initialised in stationarity, following burn-in, and then run for the same number of iterations, n_s. We then calculated the effective sample size $ESS = n_s(1 + 2\sum_{k=1}^{\infty} \rho(k))^{-1}$, where $\rho(k)$ denotes the sample correlation at lag k, and where the sum was truncated at lag M if $\rho(M+1) < 0.05$. We assigned independent $Exp(10^{-4})$ prior distributions to both β and γ.

Figure 10 shows the effective sample size per second for DA-MCMC and PBLA for exponential infectious periods and Erlang infectious periods with shape parameters $m = 2, 5$. In each case PBLA eventually outperforms DA-MCMC as N increases, although the improvement is less marked as m increases, due to the increasing cost of computing the PBLA likelihood.

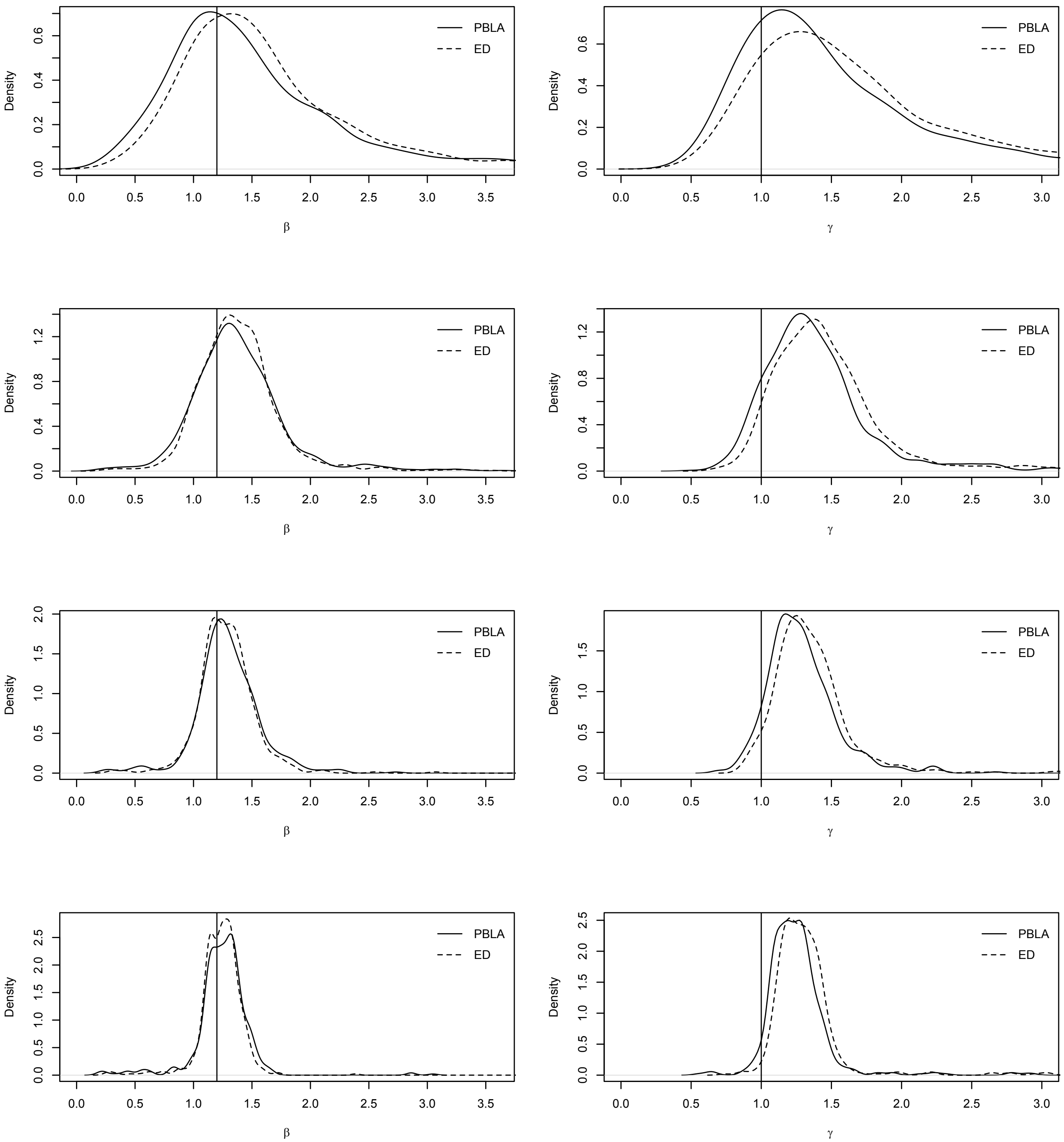

Figure 5: Comparison of maximum likelihood estimates as N varies, using simulated data sets with Erlang infectious periods and $\beta = 1.2$, $\gamma = 1$, $m = 2$, $R_0 = 2.4$. From top to bottom panels: $N = 15$, $N = 100$, $N = 250$, $N = 500$. Vertical lines show the true values.

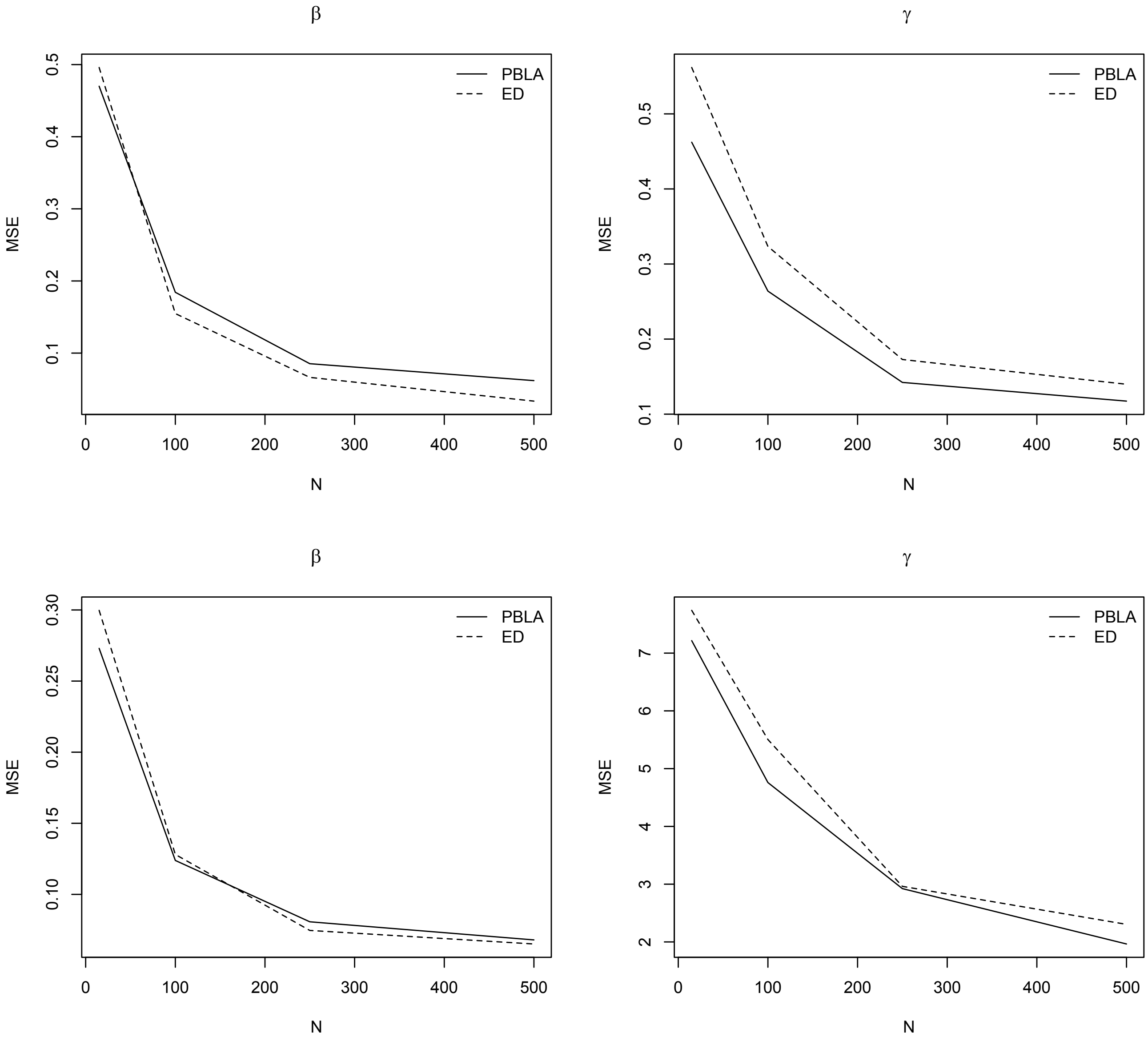

Figure 6: Mean square error (MSE) values as N varies, using simulated data sets with Erlang infectious periods and $\beta = 1.2$, $\gamma = 1$, $m = 2$, $R_0 = 2.4$.

2.4 Comparison of alternative approximations

The main text describes five possible likelihood approximations, namely (i) the standard approximation (section 3.2), (ii) using f_i for expectations (3.3.1), (iii) separating all χ and ψ terms (3.3.2),(iv) approximating the product of ψ terms (3.4.2) and (v) use of the central limit theorem (3.4.2). Note that the last two approximations were developed for the special case of the general stochastic epidemic whereas the first three are more general.

Our numerical experiments showed that the standard approximation generally performed best, although approximations (iv) and (v) are less numerically intensive. Figure 11 shows typical results based on 1000 simulations for the general stochastic epidemic model. Two key observations are that

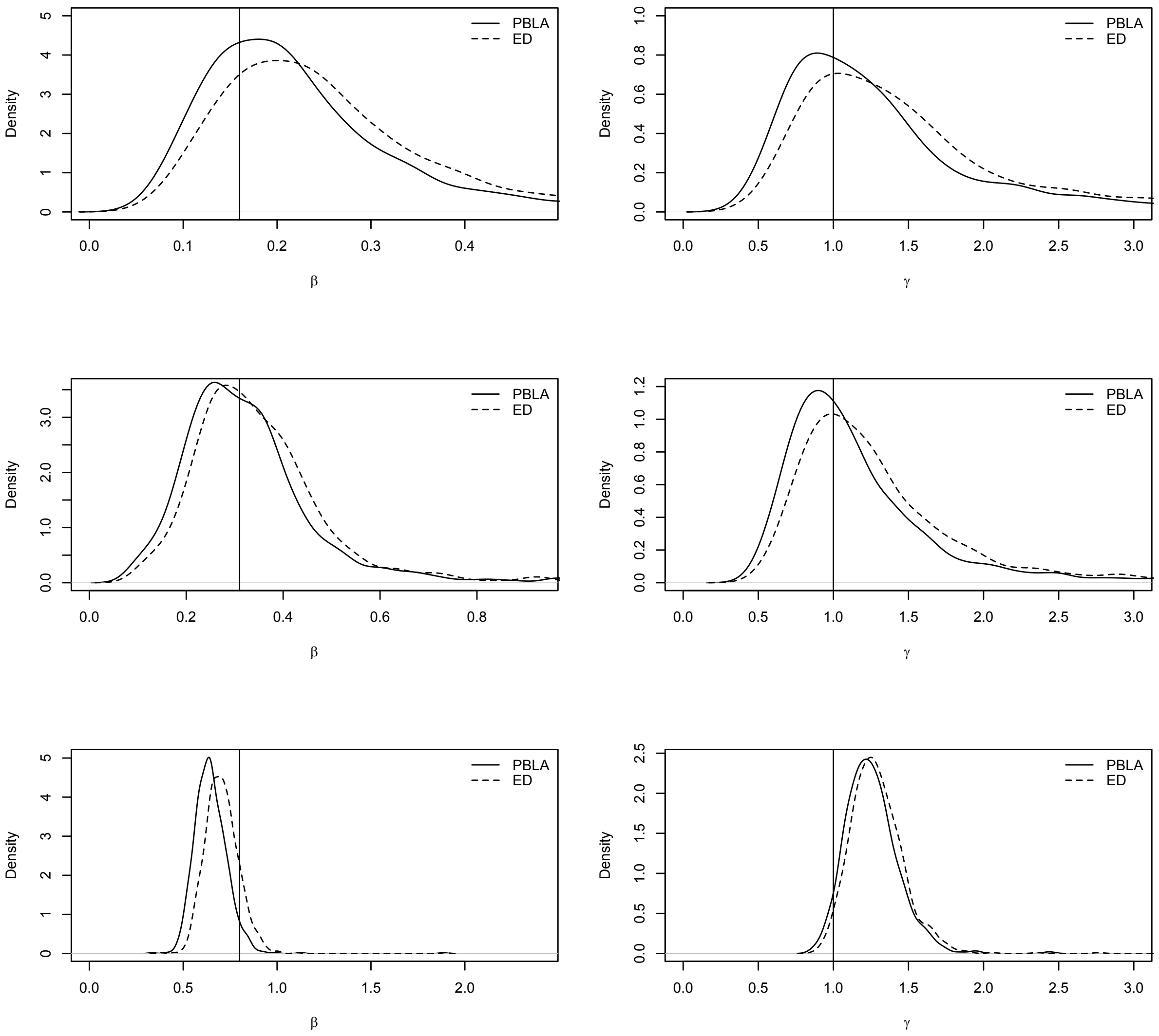

Figure 7: Comparison of maximum likelihood estimates as R_0 varies, using simulated data sets with Erlang infectious periods and $N = 80$, $\gamma = 1$, $m = 5$. Top panel: $\beta = 0.16$, $R_0 = 0.8$. Middle panel: $\beta = 0.31$, $R_0 = 1.55$. Bottom panel: $\beta = 0.8$, $R_0 = 2$. Vertical lines show the true values.

the f_i expectation method is generally inferior to the others, and that the central limit theorem approximation improves as N increases, as expected.

References

Andersson, H. and T. Britton (2000). *Stochastic Epidemic Models and their Statistical Analysis*, Volume 4. New York: Springer.

Barbour, A. D. and G. K. Eagleson (1985). Multiple comparisons and sums of dissociated random variables. *Advances in Applied Probability 17*(1), 147–162.

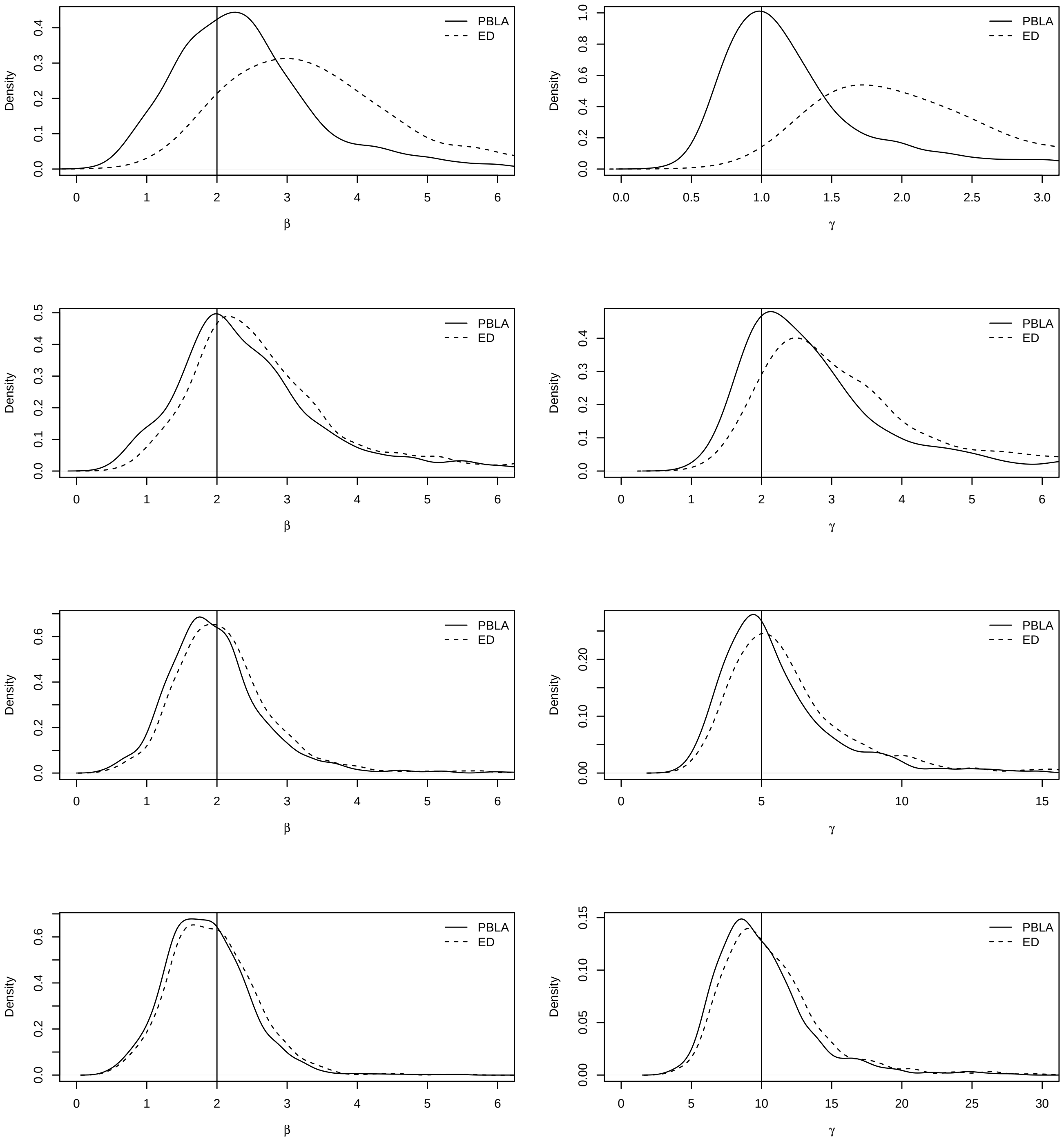

Figure 8: Comparison of maximum likelihood estimates as m varies, using simulated data sets with Erlang infectious periods and $N = 50$, $\beta = 1$, $R_0 = 2$. From top to bottom: $\gamma = m = 1, 2, 5, 10$. Vertical lines show the true values.

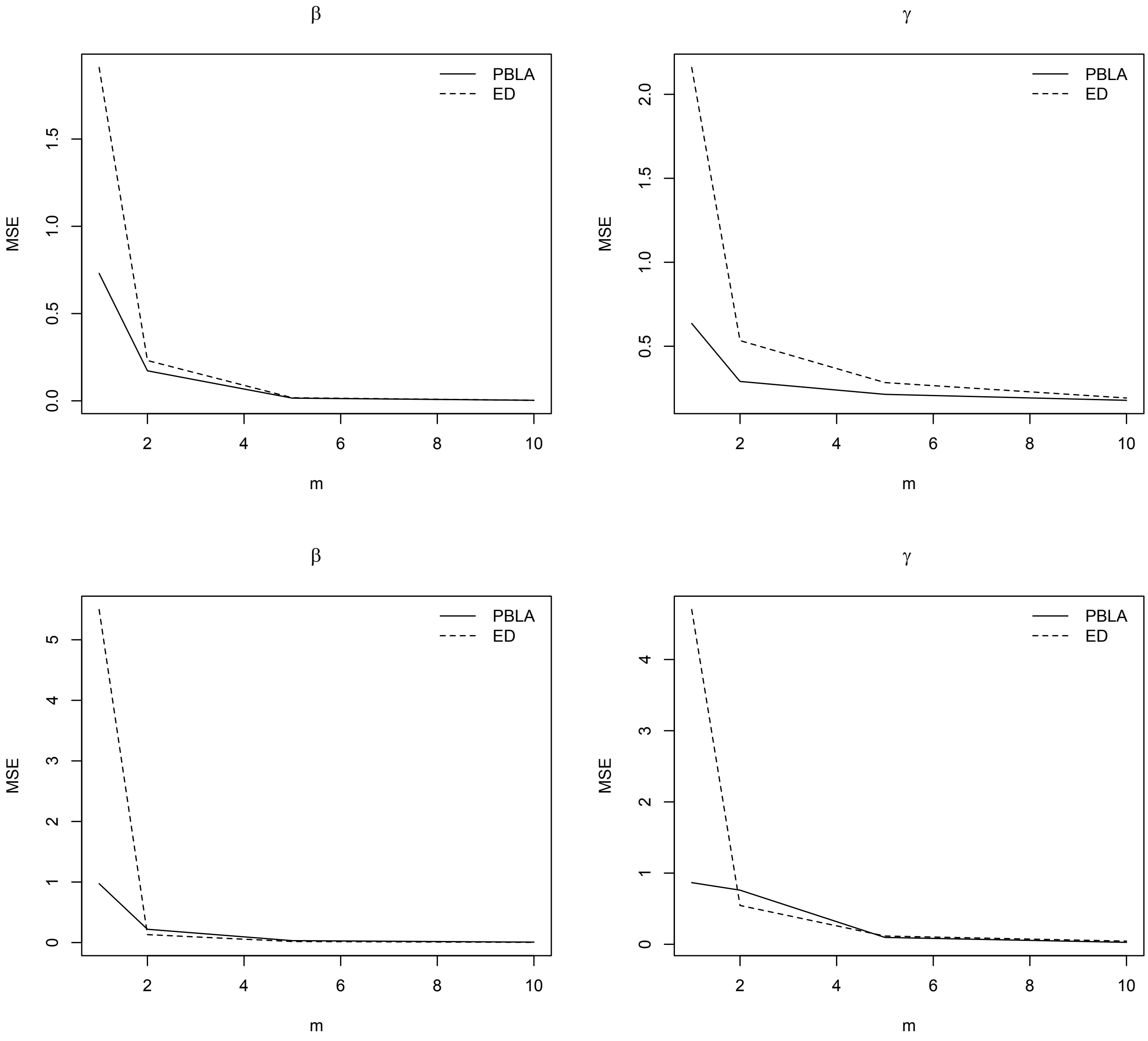

Figure 9: MSE values as m varies, using simulated data sets with Erlang infectious periods and $N = 50$, $\beta = 1$, $R_0 = 2$.

O'Neill, P. D. and G. O. Roberts (1999). Bayesian inference for partially observed stochastic epidemics. *Journal of the Royal Statistical Society: Series A (Statistics in Society) 162*(1), 121–129.

Stockdale, J. E. (2018). *Bayesian Computational Methods for Stochastic Epidemics.* Ph. D. thesis, University of Nottingham.

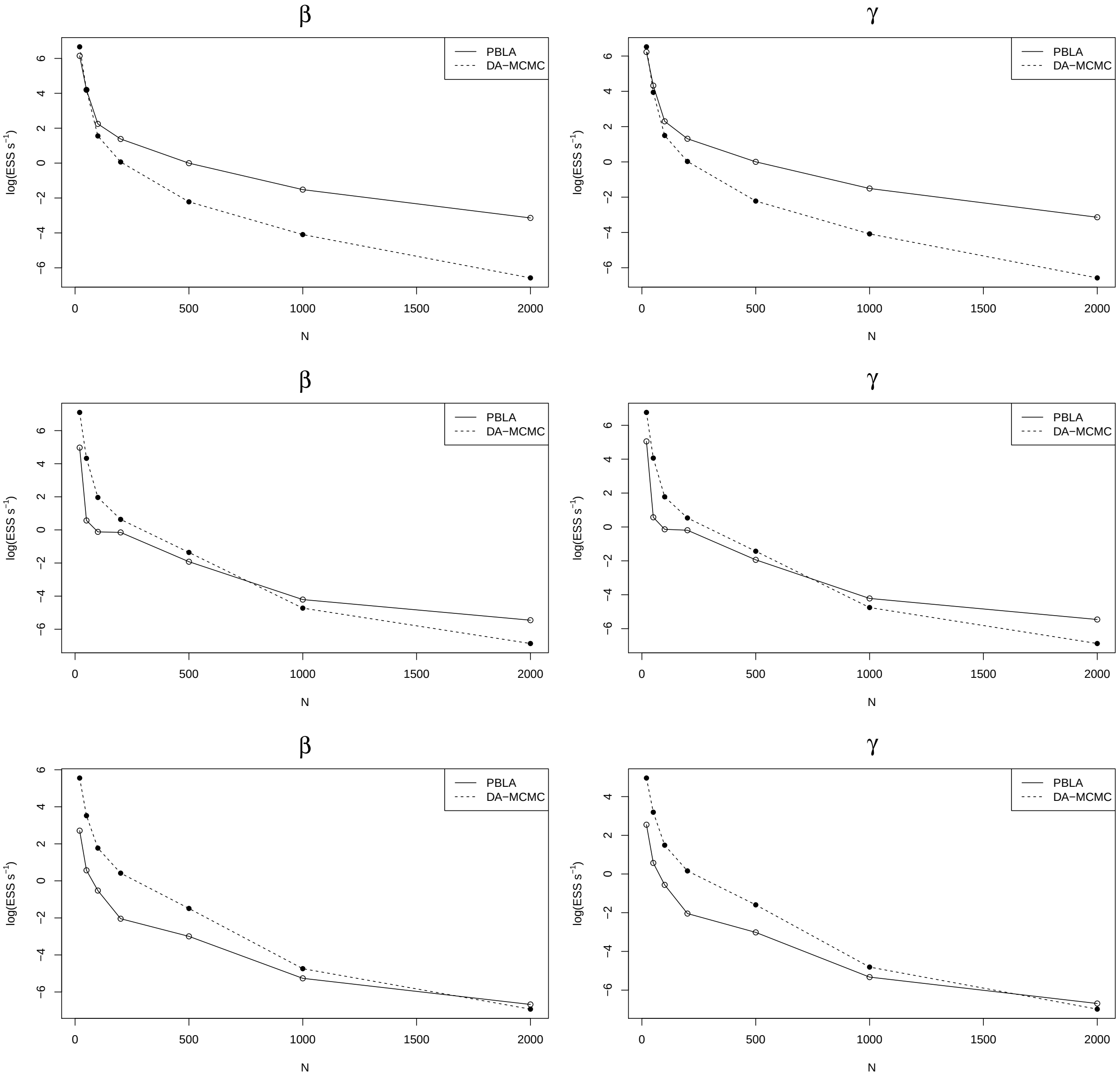

Figure 10: Log effective sample size per second of PBLA and DA-MCMC algorithms as N varies for exponential (top panels), Erlang shape $m = 2$ (middle panels) and Erlang shape $m = 5$ (bottom panels) infectious period distributions.

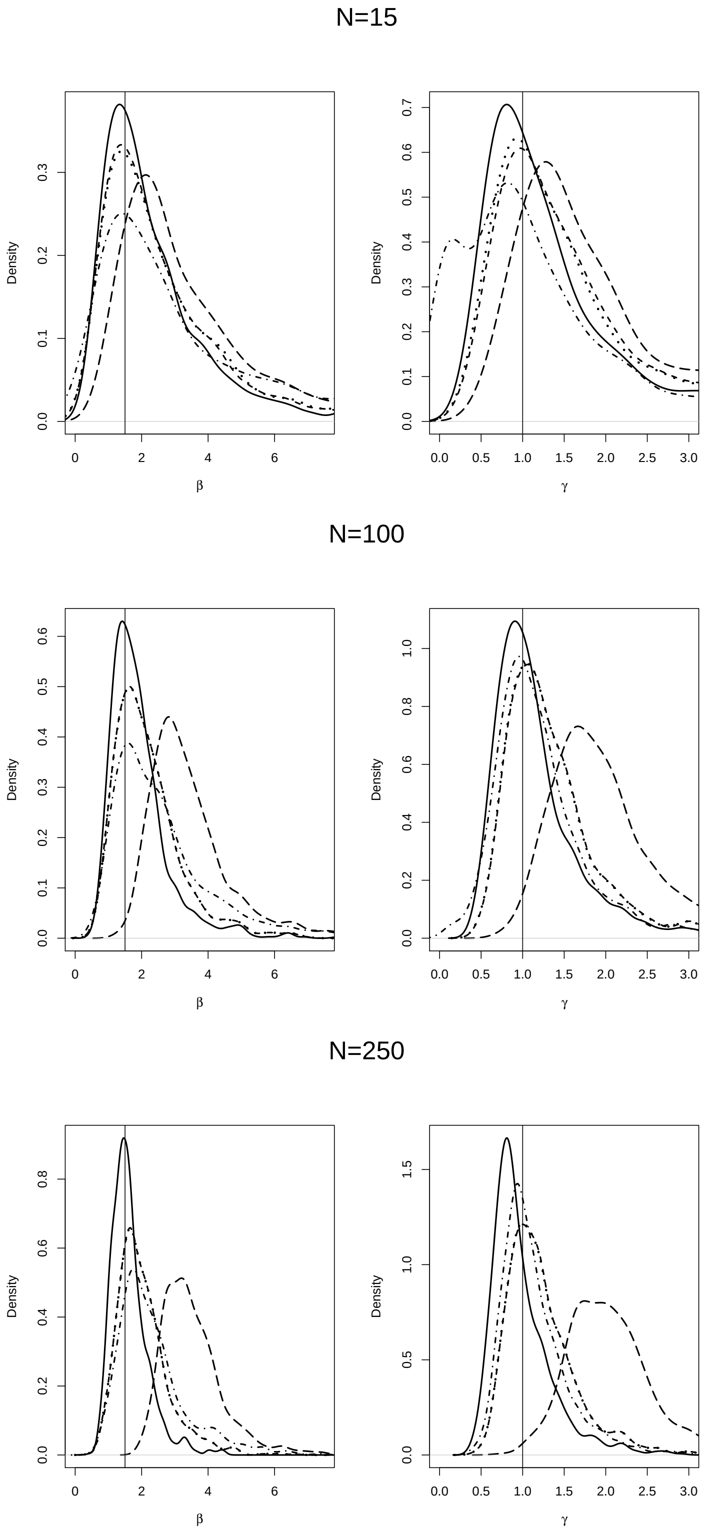

Figure 11: Comparison of different approximations. Plots show maximum likelihood estimates with $\beta = 1.5$ and $\gamma = 1$ for 1000 simulations. Solid line = standard, long dashes = f_i expectations, dots = $\chi - \psi$ separate, short dashes = ψ product and dot-dashes = central limit theorem. Vertical lines show the true values.
